# A Benchmarking Framework for Real-Time 3D Pose Estimation in Motor Rehabilitation: Development and Proof-of-Concept Study

**DOI:** 10.2196/91360

**Published:** 2026-11-09

**Authors:** Emanuel Alexander Lorenz, Beatrix Vereijken, Kerstin Bach, Xiaomeng Su

**Affiliations:** 1 Department of Computer Science, Faculty of Information Technology and Electrical Engineering, Norwegian University of Science and Technology, Sem Sælands vei 9, Trondheim, Trøndelag, 7034, Norway, +47 41353626; 2 Department of Neuromedicine and Movement Science, Faculty of Medicine and Health Sciences, Norwegian University of Science and Technology, Trondheim, Trøndelag, Norway

**Keywords:** motion capture, human pose estimation, motor rehabilitation, benchmark, skeleton morphing

## Abstract

**Background:**

Accurate motor assessment is crucial for diagnosing and monitoring neurological and musculoskeletal disorders. However, traditional manual and semiautomated methods can be subjective, time-consuming, or expensive. Real-time 3D human pose estimation (HPE) is emerging as a low-cost, noninvasive alternative. However, its clinical adoption is limited due to a lack of thorough, standardized evaluation of its accuracy and robustness in real-world clinical settings.

**Objective:**

This study aims to introduce a technical pipeline for evaluating the applicability of real-time 3D HPE in simulated clinical rehabilitation settings.

**Methods:**

The framework facilitates the evaluation of an HPE model’s spatial and spatiotemporal accuracy, real-time capability, and robustness against simulated common setup errors and camera position variations, using a marker-based motion capture system as a gold-standard baseline. To address the technical challenge of interdataset comparison, the framework includes a morphing model that harmonizes differing joint definitions.

**Results:**

Applying the framework to a popular, accessible real-time algorithm (MediaPipe) on 4 healthy participants (N=127,828 frames) showed that simulated underexposure and defocus blur had negligible effects (rank-biserial correlation [RBC] ≤0.02), whereas body segment occlusion (mean-per-joint angular error [MPJAE] increase of 1.5, SD 10 degrees; +24.3%) and insufficient multiocular calibration (MPJAE increase of 7.8, SD 14 degrees; +123.4%, RBC=0.95) significantly degraded tracking accuracy. The framework also showed significant sensitivity to nonfrontal camera placements, both side- and back-facing views (RBC 0.41‐0.49 and bias up to 2.2 degrees/37.1%), relative to stable frontal placements (RBC ≤0.03). The skeleton morphing module reduced Procrustes-aligned mean per joint position error (PA-MPJPE) by 55.29 mm (N=79,423 frames, 57.2% reduction) and its SD by 24.69 mm (N=79,423 frames, 50.7%).

**Conclusions:**

This study establishes a technical foundation for the standardized evaluation of markerless tracking for clinical applications, like motor rehabilitation. The morphing model enables interdataset comparison, and the proposed framework facilitates domain-specific interpretation of HPE performance. While further validation on pathological populations is required, this work provides the necessary engineering foundation for future validation studies.

## Introduction

### Background

In neurological and musculoskeletal disorders, accurate assessment of a patient’s motor abilities is crucial for both initial diagnosis and subsequent monitoring of progress [1]. Nevertheless, traditional manual and semiautomated assessments have limitations that hinder frequent and extensive assessments [2-4]. To overcome these limitations, new deep learning methods, including human pose estimation (HPE), are being developed for real-time, 3D evaluation of motor function in both clinical and nonclinical settings. However, as a new technology, its applicability for motor assessment in clinical settings has not yet been thoroughly evaluated. Thus, its adoption to date has been limited [5]. To address these concerns, we introduce a comprehensive framework for benchmarking HPE algorithms in various simulated clinical scenarios to evaluate not only their spatial and spatiotemporal accuracy but also their robustness to common setup errors and variabilities. Unlike existing 3D HPE benchmarks, which evaluate accuracy on curated datasets under controlled laboratory conditions, our framework targets the specific requirements of motor rehabilitation, shifting the evaluation from theoretical accuracy on curated datasets toward clinical deployability. Its specific contributions are listed at the end of this section. We explicitly frame this study as a technical proof-of-concept in healthy participants to establish the evaluation pipeline and identify necessary methodological considerations for future clinical validation.

### Clinical Motor Assessment

Traditionally, motor assessments largely depend on the manual evaluation of specific movements and tasks via standardized tests, which are well established for different pathologies and populations [3,6]. While manual assessments allow for personalization and increased accessibility, even in resource-limited settings, they are often time-consuming, subjective, and dependent on the presence of experienced personnel [2,3,7].

Thus, semiautomated assessments have been developed to address these limitations, offering objective, precise, and real-time measurements using optical systems (eg, marker-based motion capture systems for assessing movement quality in weight-shifting exercises [8]), mechanical systems (eg, pressure sensors for gait analysis [9] and inertial measurement units for upper limb assessment [10]), and robotic systems (eg, exoskeletons for assessing reaching movements [11]). However, despite their advantages, they are often expensive, intrusive in their application, and also require trained personnel [4]. Additionally, they may suffer from artifacts caused by biological and environmental variables [12,13] or by sensor placement errors [14], which can affect their accuracy and reliability.

### HPE in Motor Assessment

Recent advances in machine learning have enabled markerless joint position tracking from generic image data using HPE. Thus, low-cost camera systems can be used in diverse environments to track movements in real time. Consequently, HPE has emerged as a technology for clinical and nonclinical applications, enabling continuous, noninvasive monitoring of motor function, including gait, balance, and range of motion [5]. The field has advanced rapidly in recent years. Transformer-based lifters such as MotionBERT [14] elevate 2D pose sequences to 3D by modeling spatiotemporal joint dependencies, while one-stage real-time detectors such as RTMPose [15] and the YOLO-Pose family [16] jointly detect people and regress keypoints at high frame rates. Against this backdrop, MediaPipe is best characterized not as the most accurate model, but as a widely used, well-documented, and readily deployable one: comparative gait studies report that earlier models such as OpenPose can match or exceed it on clinically relevant joints [17], and its former advantages in speed and ease of use are increasingly shared by newer lightweight architectures. Although current offline systems achieve angular errors (AEs) below the maximum permissible AE of 5 degrees for clinical applications [18,19], they cannot provide immediate feedback during rehabilitation exercises. This limits their applicability for real-time correction and adaptation of movement patterns during activities such as exergaming [20]. Online systems, however, do not yet achieve the same spatial accuracy [21]. Furthermore, HPE still struggles with real-world environments, diverse clothing, and setup errors, with occlusion now the principal remaining challenge; recent architectures address it explicitly, for example, through diffusion- and transformer-based designs robust to occluded and noisy keypoints [22], though it is not yet fully solved [5,18,23].

While some HPE approaches use red, green, blue-depth (RGB-D) sensors, standard RGB-based HPE relies solely on 2D image data, with no depth information provided directly to the model. However, while state-of-the-art models can infer 3D poses from a single 2D image frame, this remains an ambiguous problem, as a single 2D pose representation can correspond to multiple 3D representations [24]. Thus, multiocular systems, which capture data from various camera angles, are still advised, as triangulating 2D poses across different viewpoints can improve the accuracy, reliability, and robustness of 3D reconstructions by providing additional information. However, the benefits of multiocular setups are associated with increased system complexity and higher computational demands [5].

### Benchmarking of HPE Models

The adoption of HPE in clinical rehabilitation depends on its accuracy, reliability, and robustness [5]. Health care providers are cautious about adopting new technologies due to concerns about patient safety, data privacy, and resource reallocation [25]. While general evaluation pipelines for HPE models exist, such as MMPose [26], PoseTrack [27], and other comparative analyses [24], they primarily evaluate HPE models’ accuracy on generic movements and metrics like mean-per-joint position error (MPJPE) and percentage of correct keypoints [28]. While these metrics are valuable for benchmarking HPE models, they lack clinical relevance, particularly for rehabilitation applications that demand higher precision, specifically for angular changes and contextual robustness.

Recent studies have partially addressed these gaps by including clinically relevant evaluation metrics, such as joint angle errors [18,29-32], testing HPE models on clinically relevant movements [29,31,32], or evaluating the impact of different setups and errors [18,33]. The closest systematic efforts, Rode et al [34], who benchmarked 11 monocular estimators against marker-based capture, and Wade et al [35], who reviewed markerless methods for clinical gait, either compare accuracy under controlled conditions or survey limitations qualitatively. None combine simulated clinical setup errors, per-condition robustness reporting, and cross-dataset joint harmonization in a single reproducible pipeline [5].

### Datasets for HPE in Rehabilitation

Deep neural networks, like HPE models, must be trained on large, diverse, and precisely annotated datasets to provide accurate and robust joint locations across various settings and populations. Therefore, selecting appropriate datasets for training and evaluation is critical [36].

Over the past decade, several well-known datasets, such as Human3.6M [37], MPII [38], and COCO [39], have been released. However, these datasets focus on everyday activities within controlled settings and do not include rehabilitation-specific movements. In contrast, more recent clinically relevant datasets incorporate domain-specific movements and participant groups, thereby better capturing the complexities encountered in rehabilitation settings [5,40-42]. However, these clinical datasets are suboptimal for domain-specific HPE training; they either lack full-body joint coverage despite high spatial accuracy (eg, UCO [40]) or rely on lower-precision baselines limited to specific pathologies (eg, KIMORE [41] and FineRehab [42]), failing to provide a comprehensive, high-accuracy standard for general motor rehabilitation.

Furthermore, HPE training datasets often exhibit inconsistencies in joint center definitions due to varying annotation protocols, such as manual labeling versus marker-based derivation, variations in surface marker placement, and the specific biomechanical models applied. This variation stems from the differing methods used to establish ground-truth joint center locations, such as manual annotations or biomechanical models [37-39]. As a result, these discrepancies lead to inconsistencies in the estimated keypoints when training or evaluating on alternative datasets [23,36]. To address this, several strategies have been developed to align joint center locations across datasets. Rapczyński et al [23] applied linear transformations and scale normalization, while Wandt et al [43] introduced a 2D skeleton-morphing network that aligns keypoints using residual blocks. More recently, Sárándi et al [36] used an affine-combining autoencoder to compress 3D joint keypoints from different datasets into a smaller set of latent keypoints, preserving the geometric relationships across datasets. Thus, these alignment methods enable consistent integration of heterogeneous datasets, improving HPE model comparability.

### Research Objectives

This study aims to develop and validate a framework for evaluating the applicability of real-time 3D HPE algorithms in simulated clinical rehabilitation settings, benchmarking an exemplary model against a marker-based gold standard after aligning keypoint definitions. We address 2 research questions (RQs):

- RQ1: How can a comprehensive framework be designed to evaluate the spatial and spatiotemporal accuracy of a real-time 3D HPE algorithm for clinical motor rehabilitation?
- RQ2: How does the proposed framework assess the robustness of HPE algorithms against varied camera angles, common setup errors, and clinically relevant movements?

### Contributions

This work makes the following contributions: (1) a modular benchmarking framework that evaluates real-time 3D HPE models under simulated clinical conditions, integrating environmental augmentations, and a skeleton morphing module for cross-dataset comparability; (2) a set of clinically motivated spatial and spatiotemporal metrics reported per joint and body segment; (3) an empirical benchmark of a widely used, accessible HPE model (MediaPipe) across camera configurations, movements, and simulated setup errors; and (4) a validation of the morphing module against controlled synthetic offsets, showing that it corrects systematic joint-definition differences without suppressing tracking error.

## Methods

### Benchmarking Framework Architecture

The benchmarking framework (illustrated in Figure 1) for real-time 3D HPE models augments the video input according to common errors possible in clinical settings. A modular design was chosen to address the gap between technical engineering benchmarks and actual clinical needs, while allowing for future adaptations. By integrating an augmentation module to simulate real-world setup errors and a skeleton morphing module to align conflicting dataset definitions, the framework solves the problems of a lack of environmental testing and interdataset incomparability identified in the Introduction section. This specific structure ensures that the evaluation measures true clinical robustness rather than just theoretical accuracy, helping developers to choose the most applicable HPE model and ultimately increasing trust in the technology.

It evaluates the spatial and spatiotemporal accuracy and real-time capability of both mono- and multiocular HPE models across different clinically relevant movements and body segments. The pipeline consists of four steps: (1) data augmentation, (2) pose estimation, (3) skeleton morphing, and (4) evaluation.

**Figure 1.**
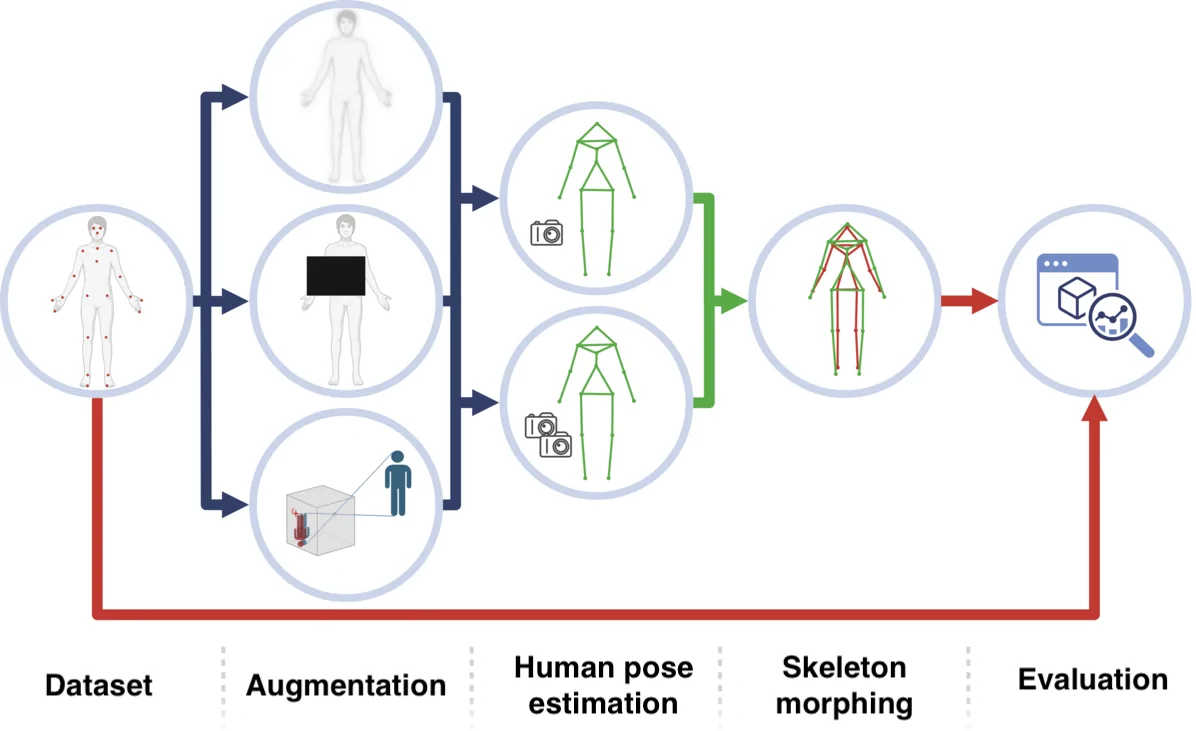
Modular benchmarking framework for the clinical validation of 3D human pose estimation. The pipeline evaluates human pose estimation performance by processing ground-truth data through 4 sequential modules, which are (1) frame augmentation: simulating clinical environmental setup errors, including defocus blur, body segment occlusion, and extrinsic camera calibration errors; (2) human pose estimation: extraction of 3D coordinates using monocular or multiocular camera configurations; (3) skeleton morphing: a dataset-specific residual neural network that harmonizes joint definitions between datasets; and (4) evaluation: calculation of domain-specific spatial and spatiotemporal metrics. Visual coding indicates data types: blue arrows represent video streams, green arrows represent keypoints in the human pose estimation-native format, and red arrows represent keypoints in the gold-standard dataset format. Image created in BioRender by EAL [44] and licensed under CC BY 4.0 [45].

### Augmentation Module

This module simulates the variability of clinical environments by augmenting video inputs and calibration data. It applies image perturbations (eg, blur, occlusion, and lighting changes) and adds noise to camera calibration and temporal synchronization. This allows us to evaluate the algorithm’s robustness under suboptimal, yet realistic, conditions.

### HPE Module

This module provides a template to incorporate any standard 3D HPE model. It supports both monocular models (inferring 3D pose from single frames) and multiocular setups (triangulating 2D keypoints from multiple views). This step generates a raw skeletal prediction based on the augmented input frames.

### Skeleton Morphing Module

As available HPE models are trained on different, at times not publicly available datasets, their definition of the anatomical location of joint center positions varies. To address the discrepancy between the surface-based keypoints of the HPE model and the internal anatomical joint centers of the marker-based gold standard, we implemented a skeleton morphing module. The skeleton morphing module is a residual neural network, based on the morphing model by Wandt et al [43], that maps the HPE keypoints onto the gold-standard (Plug-in Gait) joint-center definition. It takes the 16 predicted joints (48 values) as input, projects them to a latent representation through a fully connected layer, refines them through 2 residual blocks (fully connected layers with leaky rectified linear unit [ReLU] activation and dropout), and outputs a per-joint correction added to the input pose through a residual connection. It is trained per dataset to minimize the Procrustes-aligned mean squared error between the morphed and gold-standard skeletons, so that the correction captures the systematic offset between anatomical protocols rather than pose- or subject-specific variation. We furthermore validate the skeleton morphing module by adding linear and nonlinear offsets and noise to both the ground-truth marker-based data and MediaPipe’s pose output rather than testing a second real HPE model, because in real data the joint-definition offset is confounded with tracking error and cannot be isolated. The synthetic protocol lets us quantify offset recovery and noise preservation separately (full architecture, training, and validation in Multimedia Appendix 1).

### Evaluation Module

The final module assesses the model’s applicability for clinical rehabilitation by calculating spatial and spatiotemporal metrics (defined in Table 1) and monitoring real-time inference speed. In clinical settings, key metrics include joint positions and their trajectories, such as movement velocity. Thus, evaluating the model’s accuracy across relevant domains, such as spatial and temporal, is essential. To evaluate the intrinsic morphological correctness of the estimated pose, we aligned the estimated pose with the ground truth using Procrustes alignment [46]. While rigid coordinate system alignment would preserve the absolute trajectory information necessary, for example, for global gait parameters, Procrustes alignment isolates the algorithm’s ability to reconstruct the internal body geometry by normalizing for translation, rotation, and scale. This allows for a focused assessment of the algorithm’s relative joint angle accuracy, crucial for a wide variety of stationary clinical assessments.

**Table 1.** Metrics for evaluating the human pose estimation model’s spatial accuracy, spatiotemporal accuracy, and inference time. The metrics were chosen to facilitate clinical evaluation across relevant domains for rehabilitation applications. Their respective equations can be found in Multimedia Appendix 1.

Metric	Description	Unit
Spatial accuracy		
MPJPE^a^	The MPJPE calculates the Euclidean distance between the predicted keypoints and their respective ground truth over all frames and joints to evaluate the spatial accuracy. Lower is better.	mm
MPJAE^b^	The MPJAE calculates the average deviation between predicted and the ground truth joint angles across all axes and joints. The respective angles are calculated as the deviation from the T-pose [47]. Lower is better.	degrees
Spatiotemporal accuracy		
MPJAVE^c^	The MPJAVE calculates the average deviation between the smoothed (Savitzky-Golay filter, $windowlength=2\frac{1}{T}+1samples$, polynomialorder=3) predicted joint angle and ground truth over a time window, based on the temporal derivative of the joint angle. Lower is better.	°/s
Pearson *r*	The mean-per-joint Pearson correlation coefficient (*r*) measures spatiotemporal similarity between the predicted joint trajectories and ground truth. Closer to 1 is better.	1
Real-time capability		
Inference time	Inference time reflects the model’s real-time applicability averaged over all frames. Lower is better.	ms

a MPJPE: mean-per-joint position error.

b MPJAE: mean-per-joint angular error.

c MPJAVE: mean-per-joint angular velocity error.

It should be noted that although the coefficient of multiple correlation has been recommended to assess the similarity of waveforms because it accounts for baseline offset and scaling differences [48], this study uses the Pearson correlation coefficient (*r*). Since the applied morphing model compensates for intersensor and dataset discrepancies in baseline and scale, their effects are negligible.

### Proof-of-Concept Implementation

To demonstrate the applicability of the proposed framework, we conducted a proof-of-concept validation study using a popular, accessible real-time HPE algorithm (MediaPipe) and a domain-specific dataset.

### Dataset and Experimental Protocol

The dataset, which served as the study’s ground truth, was acquired using a marker‐based motion capture system (Qualisys AB) hardware, synchronized via transistor-transistor logic (TTL) with RGB video recordings from 6 camera angles (FLIR Systems Inc.). Nineteen healthy participants (mean age 30, SD 8.2 years; mean BMI 25, SD 3.7 kg/m²; 9 females) were recorded. Fifteen participants were used to train and test the skeleton morphing model and 4 for the subsequent benchmarking framework itself. Detailed acquisition, synchronization, and processing protocols are provided in Multimedia Appendix 1.

The movement protocol was designed in collaboration with subject matter experts in physiotherapy and movement science to represent common motor rehabilitation tasks. The protocol included 10 repetitions of left-sided planar upper- and lower-body movements and whole-body functional tasks (eg, sit-to-stand). A detailed list of movement sequences is provided in Multimedia Appendix 1.

### Skeleton Morphing Model Training

The model was trained using leave-one-out cross-validation and tested on a subset of the VisLab dataset, consisting of 15 participants (6 females), with a mean age of 32 (SD 11) years, a mean height of 168 (SD 10) cm, and a mean weight of 75 (SD 13) kg. Leave-one-out cross-validation was used to maximize training data usage while ensuring the model learned a generalized mapping of the joints instead of memorizing subject-specific anthropometry. These participants were excluded from the subsequent benchmarking framework. Detailed information on the model architecture and training is provided in Multimedia Appendix 1.

### HPE Model Selection

To demonstrate the functionality of our framework, the commonly used real-time 3D HPE algorithm MediaPipe (Pose Landmarker Full) was chosen. As a lightweight, single-stage model optimized for accessibility and real-time use rather than maximal accuracy, MediaPipe is not the most accurate HPE model available [49]. We selected it as a representative, readily deployable baseline, and the framework itself is model agnostic. MediaPipe’s 3D world coordinates were used for monocular pose estimation, while for multiocular pose estimation, MediaPipe’s 2D image coordinates were triangulated via direct linear transformation (DLT) [50]. The inference was performed on an AMD Ryzen 9 5950X CPU with 64 GB RAM and an NVIDIA GeForce RTX 3090 GPU.

### Simulation of Setup and Environmental Errors

For the simulation of common errors in clinical environments, the image frames were augmented using the Python library imgaug [51] and pixlib [52]. Unfocused camera setups were simulated using strong Gaussian blur, while dim ambient lighting was replicated through exposure reduction, achieved by blending frames with a black background at 15% opacity. To simulate environmental obstructions, random occlusions covering 10% to 25% of the image size were superimposed onto the central region, and the uniform laboratory background was replaced with synthetic noise. Furthermore, for multiocular setups, custom scripts were used to perturb the extrinsic calibration parameters by adding Gaussian noise to the projection matrix (resulting in an approximate 2.5-pixel reprojection error) and to introduce temporal desynchronization between camera streams by randomly offsetting frames by 0 to 1 frame samples. A visualization of these augmentations can be found in the Multimedia Appendix 1.

In addition to the named error sources, the effect of different camera placements was tested. The cameras’ respective placements are shown in Figure 2, and the camera combinations used for multiocular setups were front left (fl), front right (fr); fl, back left (bl); front middle (fm), side left (sl); fl, fm, fr; fl, fm, sl; and fl, fm, bl, sl. The specific camera positions and their height (1m) were selected to represent typical spatial constraints in clinical environments. For instance, the “fm” and “sl” positions simulate common single-tripod setups, while corner-mounted configurations (eg, fl, fr) reflect permanent installations in rehabilitation gyms where floor space must remain unobstructed.

**Figure 2.**
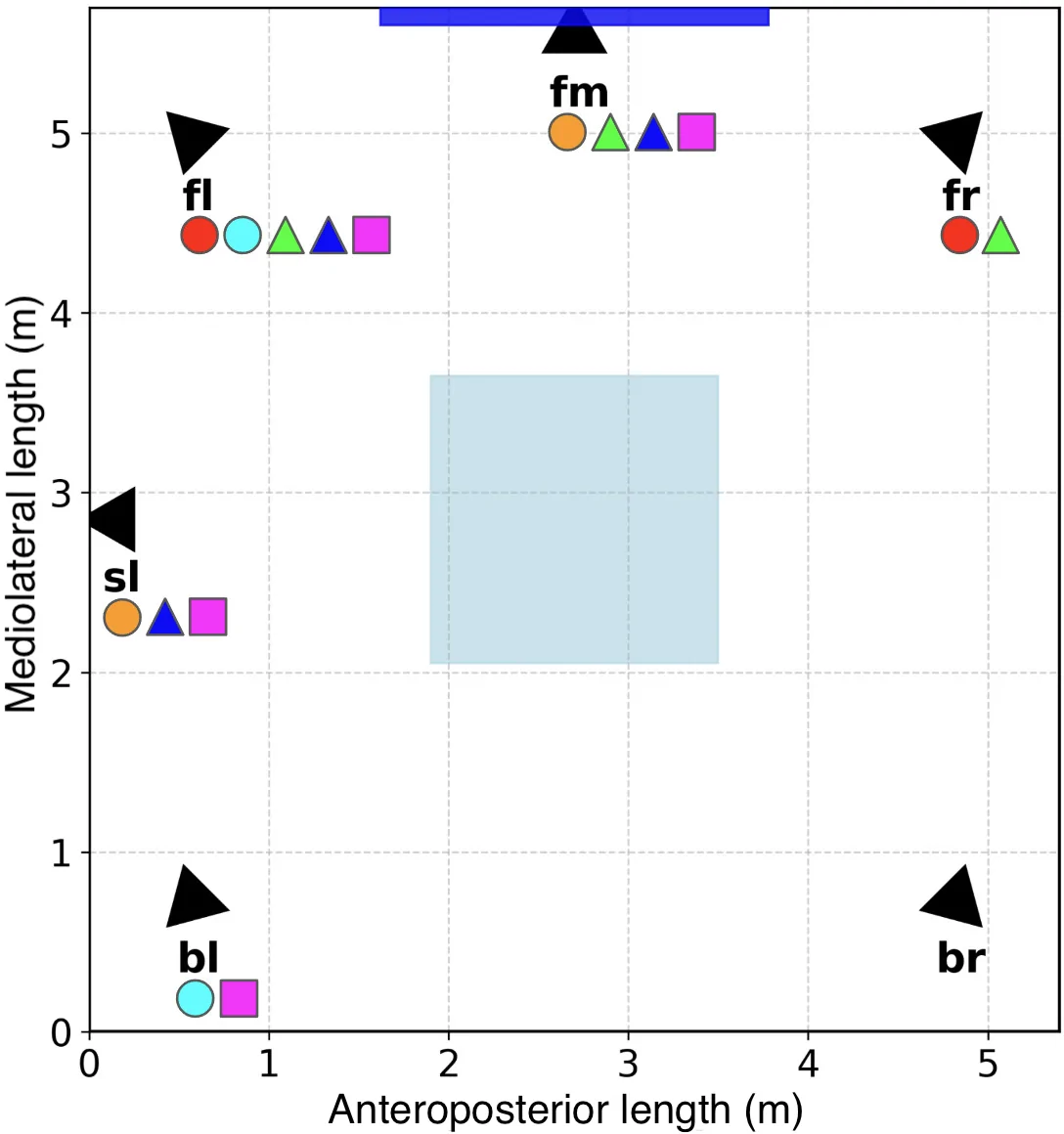
Top-down schematic of the VisLab laboratory setup, illustrating red-green-blue video camera positions and the movement area. The setup was used to acquire the gold-standard motion capture data from 19 healthy participants. Colored symbols below camera IDs indicate the 2-camera (dots), 3-camera (triangles), and 4-camera (rectangles) multiocular setups tested. Bl: back left; br: back right; fl: front left; fm: front middle; fr: front right; sl: side left.

### Statistical Analysis of Evaluation

The evaluation pipeline results are based on a subset of the VisLab dataset, which includes 4 participants (2 females) with a mean age of 29 (SD 4) years, a mean height of 170 (SD 12) cm, and a mean weight of 73 (SD 13) kg.

Nonnormality in mean-per-joint angular error (MPJAE), the main evaluation metric, was confirmed using Q-Q plots. The Wilcoxon signed-rank test was used to compare camera positions and augmentations across all movement conditions. To control for false positives due to multiple comparisons, the Benjamini-Hochberg procedure was applied [53]. However, due to the high sample number (N=127,828), statistical significance tests are highly sensitive, yielding *P*<.001 even for clinically negligible differences. Therefore, the interpretation of results prioritizes effect sizes (rank-biserial correlation [RBC]) and bias over *P* values. To keep the main tables concise, they report the primary metric (MPJAE) together with its bias and effect size. Corresponding MPJPE, mean-per-joint angular velocity error (MPJAVE), and Pearson *r* values, along with tables of statistical significance, are provided in Multimedia Appendix 1. The magnitude of these differences was further assessed using the RBC as effect size. Differences between movement types were assessed using pairwise post hoc comparisons using the Games-Howell test with Benjamini-Hochberg correction to control for false positives [53,54].

### Ethical Considerations

The study was approved by the Data Protection Services of the Norwegian Agency for Shared Services in Education and Research (reference number: 878754) and is in accordance with the Declaration of Helsinki. Multimedia Appendix 1 provides additional information on the VisLab dataset.

## Results

### Impact of Keypoint Morphing on Baseline Accuracy

To ensure the framework allows for accurate interdataset comparison, we evaluated the skeleton morphing module. The morphing model decreases the overall Procrustes-aligned mean per joint position error (PA-MPJPE) by 55.29 mm (57.2%) and its SD by 24.69 mm (n=79,423, 50.7%). We observed a significant decrease in the MPJPE error across all tracked keypoints after applying the morphing model (Wilcoxon signed‐rank test, W=1783; *P*<.001), alongside a significant reduction in variability (Levene test, *F*_1, 158,844_=13,008.1; *P*<.001; Figure 3).

Joint‐wise analyses revealed significant improvements across all keypoints, with a large effect (RBC mean 0.86, SD 0.10). Notably, while the elbow, wrist, and hip joints exhibited some side‐dependent differences in effect size, the mean effect size difference between the left and right sides was minimal (RBC_left-right_=0.02).

To confirm that this improvement reflects a correction of the anatomical offset rather than a suppression of genuine tracking error, we additionally validated the morphing model against controlled synthetic offsets with known ground truth (Multimedia Appendix 1, validation method of morphing model). On held-out participants, the model achieved a mean offset recovery rate of 65.0% for movement-dependent (nonlinear) offsets, compared to 21.0% for a per-joint linear correction, while maintaining a noise-preservation ratio of 0.97-1.00, confirming that it harmonizes joint definitions without masking tracking error.

**Figure 3.**
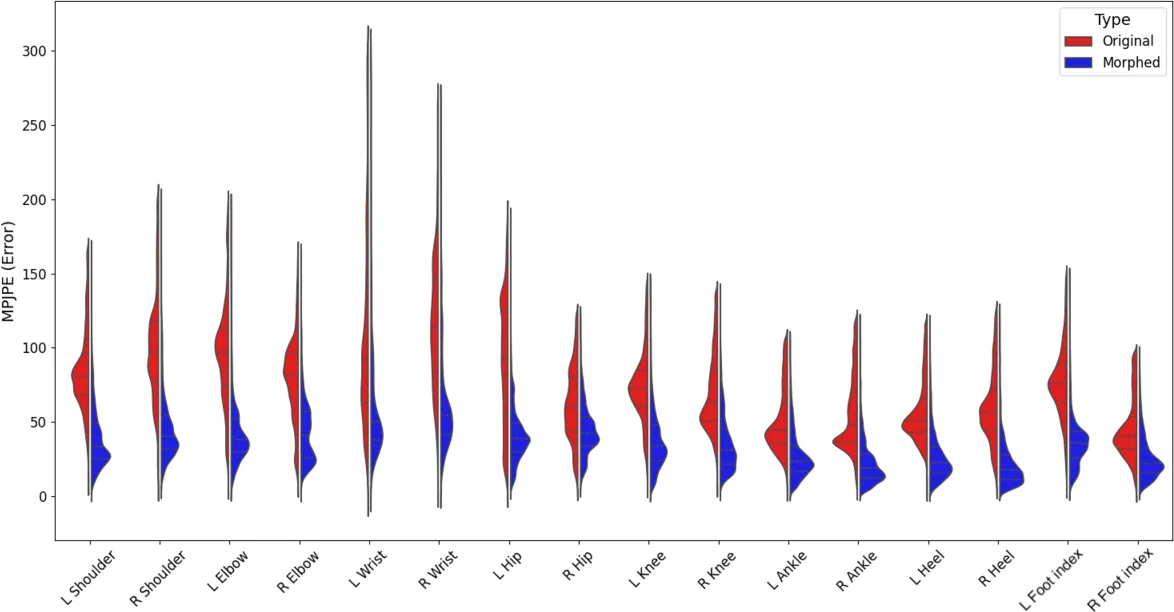
Mean-per-joint position error before and after morphing MediaPipe keypoints to the Plug-in Gait joint center positions. The morphing model was trained on 15 healthy participants to improve cross-dataset comparison validity. L: left; MPJPE: mean-per-joint position error; R: right.

### Joint-Specific Sensitivity Analysis

The framework’s joint-wise resolution identified anatomical inconsistencies of the HPE model. By evaluating AEs for each joint, we identified which joints are most prone to inaccuracies. The results are based on nonaugmented inputs for the default camera positions (mono: fm, multi: fl-fr) and all movements.

Figure 4 illustrates that the MPJAEs generally tend to be slightly higher in multiocular setups for the default camera placements. Distal upper body joints, such as the wrists and elbows, exhibit higher AEs than proximal or lower body joints in both setups. The knee and left upper body joints show a relatively high variability.

**Figure 4.**
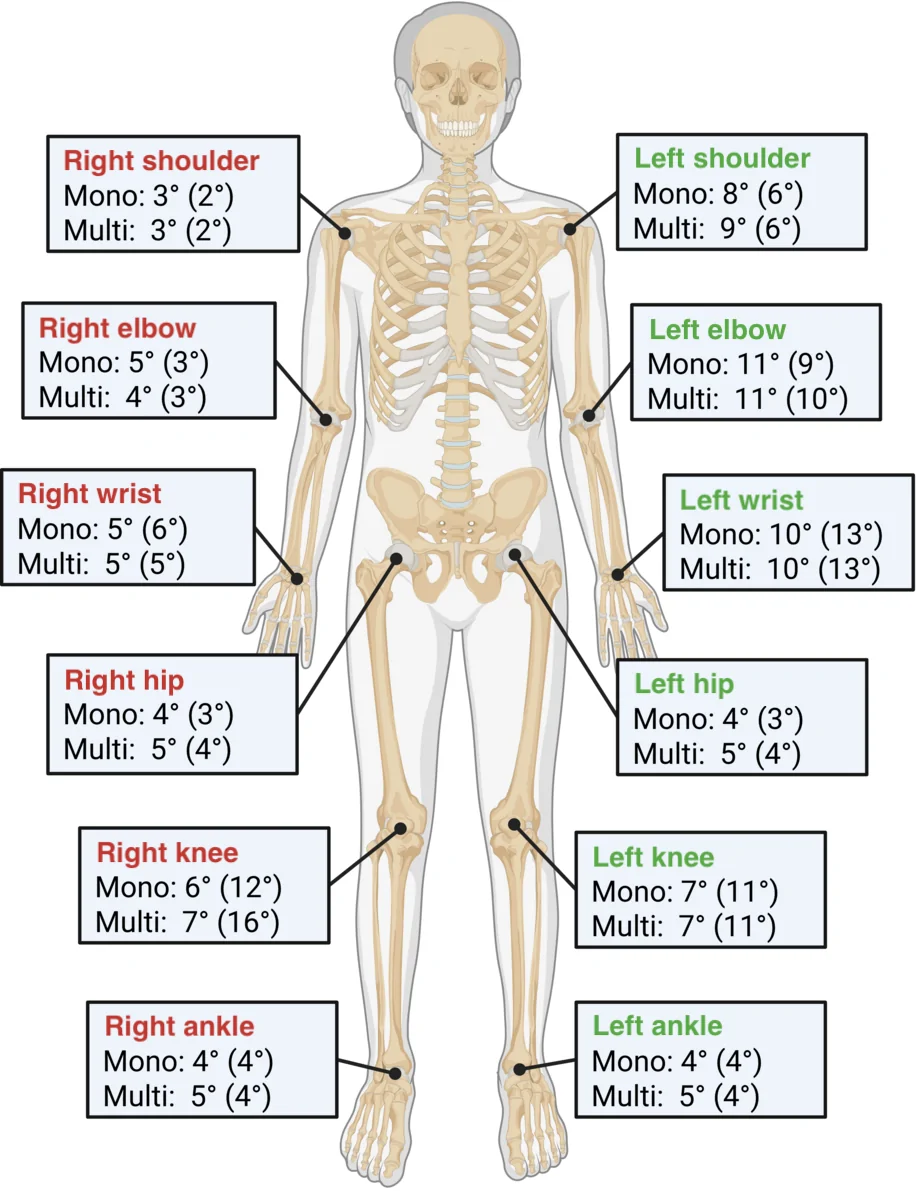
Mean-per-joint angular error and SD for each joint in monocular and multiocular human pose estimation setups. Results are based on nonaugmented inputs for the default camera positions on 4 healthy participants (N=127,828 frames). Image created in BioRender by EAL [55] and licensed under CC BY 4.0 [45]. Mono: monocular; multi: multiocular.

### Benchmarking Robustness to Simulated Clinical Setup Errors

The impact of common setup errors on measurement accuracy was assessed for the default camera positions (mono: fm; multi: fl, fr). Table 2 displays the MPJAE, its bias, and effect size across simulated setup errors, averaged over all joints and movements; corresponding MPJPE and MPJAVE distributions are shown in Figure 5.

**Table 2.** Mean (SD) values of mean-per-joint angular error across simulated setup errors for monocular and multiocular human pose estimation setups^a^.

Setup errors	MPJAE^b^ (degrees), mean (SD)		MPJAE effect size		MPJAE bias (degrees), n (%)	
	Mono	Multi	Mono	Multi	Mono	Multi
Background	6 (8)	7 (10)	0.14	0.14	0.3 (5.1)	1.0 (15.3)
Defocus	6 (8)	6 (9)	0.02	0.02	0.1 (1.3)	0.1 (1.3)
Occlusion	7 (9)	8 (10)	0.14	0.24	0.7 (12.7)	1.5 (24.3)
Underexposure	6 (8)	6 (8)	−0.02	0.02	−0.1 (−1.1)	0.0 (−0.2)
Decalibrated	—^c^	14 (14)	—	0.95	—	7.8 (123.4)
Desynchronized	—	6 (8)	—	0.06	—	0.0 (0)

a The rank-biserial correlation effect size and bias are given in comparison to the baseline (no augmentation). Results are averaged over all joints and movements using default camera positions (mono: front-middle; multi: front-left and front-right) on 4 healthy participants (N=127,828 total frames).

b Mean-per-joint angular error.

c Not applicable.

**Figure 5.**
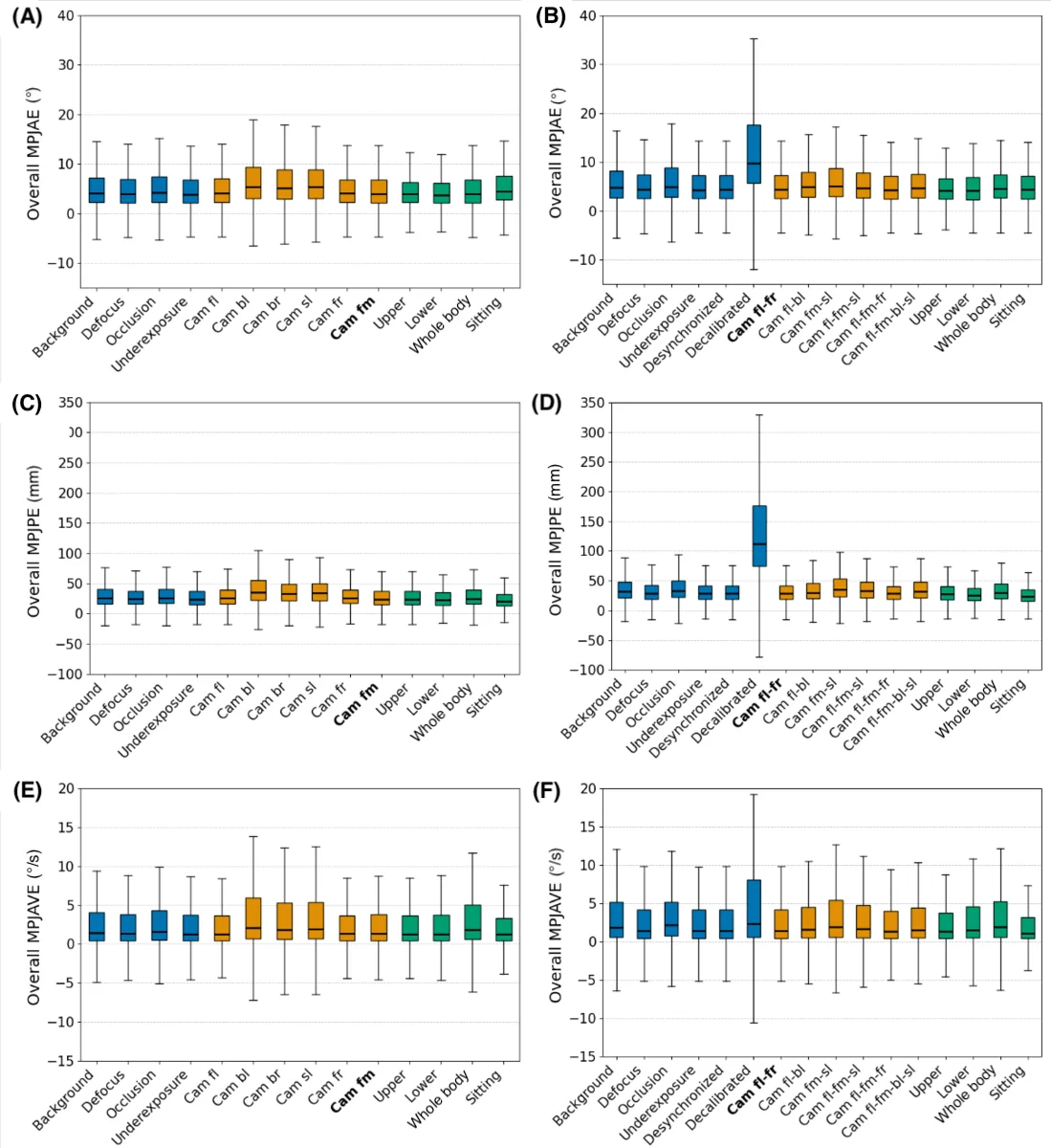
Boxplots of mean joint metrics comparing monocular and multiocular human pose estimation setups. The metrics displayed are (A and B) mean-per-joint angular error, (C and D) mean-per-joint position error, and (E and F) mean-per-joint angular velocity error. The left column (A, C, and E) represents results from the monocular setup, while the right column (B, D, and F) represents the multiocular setup. Data are grouped by setup errors, camera positions, and movement categories, summarizing the evaluation of 4 healthy participants across all simulated scenarios (N=127,828 frames). Bold markings indicate default camera positions. bl: back left; br: back right; Cam: camera; fl: front left; fm: front middle; fr: front right; MPJAE: mean-per-joint angular error; MPJAVE: mean-per-joint angular velocity error; MPJPE: mean-per-joint position error; sl: side left.

The framework verified that defocused and underexposed frames had negligible effects on measurement accuracy in both monocular and multiocular setups (RBC_defocus_=0.02 and RBC_underexposure_ between –0.02 and 0.02) and negligible biases (Bias_defocus_=0.1 degrees and Bias_underexposure_ between –0.1 degrees and 0.0 degrees) compared to the baseline condition.

Increased background noise had only a small effect (RBC=0.14) in both configurations. In the multiocular setup, the framework captured an MPJAE increase of 1 degree (15% relative increase), rising from a baseline of 6 (SD 8) degrees to 7 (SD 10) degrees and MPJPE to 45 (SD 56) mm. The MPJAVE also increased to 5 (SD 11)°/s, while the Pearson *r* decreased to 0.90. The mono‐ocular setup also remained close to the baseline, with an MPJAE of 6 (SD 8) degrees and an MPJPE of 33 (SD 27) mm.

Occlusion in the frame presented a challenge for both monocular and multiocular systems, with a small effect (RBC_mono_=0.14 and RBC_multi_=0.24). However, the metrics captured a deterioration in the multiocular setup, quantifying an MPJAE increase of 2 degrees (24.3%) to 8 (SD 10) degrees. Meanwhile, MPJPE rose to 45 (SD 53) mm, and MPJAVE reached 5 (SD 9)°/s. Temporal agreement was also affected, with the Pearson *r* dropping to 0.89.

The framework indicates a high sensitivity to extrinsic calibration of multiocular setups. By introducing a calibration error, the framework registered a large degradation (RBC=0.95), with MPJAE increasing significantly by 8 degrees (123% relative increase), rising from a baseline of 6 (SD 8) degrees to 14 (SD 14) degrees. Alongside, the MPJPE increased to 137 (SD 92) mm, and MPJAVE reached 8 (SD 16)°/s. Spatiotemporal agreement decreased considerably, with the Pearson *r* dropping to 0.65.

Finally, camera desynchronization had a negligible impact on the multiocular system (RBC=0.06 and bias=0 degrees).

### Benchmarking Robustness Across Camera Placements

The framework provided a detailed map of spatial reliability across different camera placements, allowing for the identification of optimal sensor placement. Table 3 summarizes MPJAE, its bias, and effect size for each camera placement relative to the default position (mono: fm, multi: fl-fr); corresponding MPJPE and MPJAVE distributions are shown in Figure 5.

**Table 3.** Mean (SD) of mean-per-joint angular error for all camera placements across all movements^a^.

Placement	MPJAE^b^ (degrees), mean (SD)	Effect size	Bias (degrees), n (%)
fl^c^	6 (8)	−0.01	0.0 (−0.5)
fm^d^	6 (8)	—^e^	—
fr^f^	6 (8)	0.03	0.1 (1.6)
br^g^	8 (10)	0.46	1.8 (30.2)
bl^h^	7 (9)	0.41	1.6 (28.1)
sl^i^	8 (10)	0.49	2.2 (37.1)
fl, fr	6 (8)	—	—
fl, bl	7 (8)	0.12	0.4 (6)
fm, sl	7 (9)	0.33	1.1 (16.8)
fl, fm, fr	6 (8)	−0.06	−0.2 (−3.1)
fl, fm, sl	7 (9)	0.14	0.3 (5.1)
fl, fm, bl, sl	6 (9)	0.04	0.1 (2.3)

a The rank-biserial correlation effect size and bias are given in comparison to the baseline (fm and fl,fr). Results are shown for monocular and multiocular setups on nonaugmented inputs from 4 healthy participants (N=127,828 total frames).

b Mean-per-joint angular error.

c fl: front left.

d fm: front middle.

e Not applicable.

f fr: front right.

g br: back right.

h bl: back left.

i sl: side left.

The evaluation pipeline verified that changes in camera position had only a minimal impact on pose estimation accuracy (Table 3). Across monocular setups, the metrics indicated that MPJAE remained approximately 6 (SD 8) degrees to 8 (SD 10) degrees, MPJPE ranged between 30 (SD 22) mm and 47 (SD 46) mm, and MPJAVE remained low at 4 (SD 8)°/s to 6 (SD 12)°/s, with high temporal agreement (Pearson *r*=0.92‐0.96). The framework confirmed similar stability in multiocular setups (MPJAE=6 (SD 8) degrees to 7 (SD 9) degrees, MPJPE=33 (SD 22) mm to 45 (SD 39) mm, MPJAVE=4 (SD 9)°/s to 5 (SD 11)°/s; Pearson *r=*0.93‐0.95), while quantifying that multiocular configurations were more consistent across placements compared to monocular setups.

Further, the framework indicated that differences in camera placement were statistically significant (*P*<.001). In monocular setups, the pipeline confirmed high reliability in frontal placements (fl and fr), registering negligible effect sizes (RBC=–0.01 to 0.03). In contrast, back‐facing (br and bl) and side‐facing (sl) placements yielded medium effect sizes (RBC between 0.41 and 0.49) with biases reaching up to 2.2 degrees (37.1%). For multiocular setups, most camera combinations showed negligible changes (eg, [fl, fm, fr] with RBC=–0.06 and [fl, fm, bl, sl] with RBC=0.04). Notably, stereoscopic setups with one side- or back-facing camera ([fm, sl] and [fl, bl]) exhibited a moderate effect (RBC_fm, sl_=0.33, bias_fm, sl_=1.1 degrees or 16.8%) to a small effect (RBC_fl, bl_=0.12, bias_fl, bl_=0.4 degrees or 6%).

### Influence of Movement Type on Accuracy

We evaluated the influence of various movement types—upper body, lower body, and whole-body standing and sitting movements—on measurement accuracy by analyzing their effect on the chosen metrics (Table 4 and Figure 5). The bias and effect size of the MPJAE are presented in Multimedia Appendix 1. The analysis uses only nonaugmented inputs from the default camera position.

**Table 4.** Mean (SD) of mean-per-joint angular error across different movement categories^a^.

Movement type	MPJAE^b^ (degrees), mean (SD)		Number of samples
Upper	6 (7)^c^	6 (8)^d^	30,521
Lower	6 (7)^c^	6 (7)^d^	29,818
Standing	6 (8)^c^	6 (8)^d^	37,967
Sitting	6 (9)^c^	6 (8)^d^	29,522

a Results are based on nonaugmented inputs from the default camera positions in 4 healthy participants, demonstrating the influence of movement type on pose estimation accuracy.

b Mean-per-joint angular error.

c Mono.

d Multi.

Table 4 shows that movement type only slightly influenced pose estimation accuracy. Across upper, lower, and whole-body standing and sitting movements, the metrics remained stable with an MPJAE close to 6 (SD 7-9) degrees for both monocular and multiocular setups. MPJPE varied between 25 (SD 17) mm for sitting and 36 (SD 23) mm for whole-body standing movements, while MPJAVE was consistently low, ranging from 3 (SD 8)°/s for sitting movements to 4 (SD 10)°/s across upper-, lower-, and standing-body movements. Pearson *r* (0.89‐0.95) values indicated strong temporal agreement across all movement categories. For both monocular and multiocular setups, sitting movements demonstrated the lowest errors and best temporal agreement compared to whole-body standing movements, which showed the highest spatial errors and temporal variability. Although post hoc comparisons (Multimedia Appendix 1) reveal statistically significant differences (*P*<.001) among certain movement types, the effect sizes remained largely negligible. In monocular setups, the largest observed effect size (Hedges *g*=0.24 for lower vs sitting) corresponded to only about a 1 degree difference in MPJAE. Similarly, in multiocular conditions, the largest effect size (Hedges *g*=0.16 for standing vs upper) also represented a negligible practical difference.

### Benchmarking of Real-time Capability

Last, the model’s real-time capability was evaluated by comparing processing latency across camera configurations. This analysis considers various camera counts, providing insights into each model’s performance and feasibility for real-time applications. The results are based on nonaugmented inputs for the default camera position. Processing times per frame increased incrementally with the addition of camera inputs. A single-camera setup achieved an average processing time of 23.5 (SD 0.2) ms, corresponding to a frame rate of approximately 43 fps. As complexity increased, the processing time rose to 26.3 (SD 0.7) ms for 2 cameras and 28.2 (SD 0.9) ms for 3 cameras. The 4-camera configuration resulted in the highest latency at 30.6 (SD 1) ms (~33 fps). Across all configurations (N=127,828 frames), the SD remained low, indicating highly consistent processing times regardless of the setup complexity.

## Discussion

### Principal Findings

Applying the framework to MediaPipe, we found the evaluated model robust to simulated underexposure, defocus, and background variation, but markedly degraded by body-segment occlusion, insufficient multiocular calibration, and side- or back-facing camera placements. The skeleton morphing module reduced the joint-definition offset between the HPE output and the marker-based reference (PA-MPJPE mean reduction of 55.29, SD 24.69 mm; N=79,423 frames, −57.2% reduction) while, on synthetic data, leaving injected noise intact, confirming that it harmonizes joint definitions without masking genuine tracking error. Beyond these specific results, the study’s main contribution is the evaluation pipeline itself. Because its augmentation, pose estimation, skeleton morphing, and evaluation modules are interchangeable, present or future HPE models can be assessed against the requirements of a given rehabilitation workflow. The source code for the framework and morphing model is available in GitHub [56].

### Augmentation: Validating Robustness and Defining Setup Guidelines

By simulating plausible configuration errors, the framework identifies the operational limits of evaluated HPE models and informs evidence-based setup guidelines. In this proof-of-concept, the framework indicated that underexposure, defocus, and simulated clinical backgrounds had negligible effects on the given HPE model’s performance, likely due to its training on partially augmented datasets. This is consistent with previous studies [18,21,23]. In summary, this suggests that lightweight, real-time HPE models such as the one evaluated here can tolerate the lighting and background variation tested here. This is an encouraging but preliminary indication for less controlled clinical or home environments that require confirmation on patient populations.

In contrast, occlusion remains problematic for HPE systems despite strategies such as per-point visibility classifiers [49] or integrating temporal information (eg, transformer-based models or kinematic constraints) [24]. Therefore, clinical setups must ensure that occlusions are minimized—environmental as well as self-occlusion.

Furthermore, the framework highlighted the sensitivity of multiocular setups to setup errors. While multiocular camera stream desynchronization did not degrade accuracy for slow movements, insufficient camera calibration significantly did. This highlights the need for precise calibration in multiocular setups, which is a well-known problem [57,58]. Thus, particularly for clinical applications, this resource-intensive and error-prone manual process could benefit from novel methods that facilitate automatic calibration [57] or do not require calibration [58].

Regarding camera placement, the framework can be used to indicate preferable setup configurations. In our proof-of-concept, the significant error increase in side-facing setups (>37% bias) indicates that clinical setups should prioritize frontal or corner-mounted views to minimize self-occlusion (consistent with Aguilar-Ortega et al [40]). View-dependent accuracy loss in markerless capture is well documented, particularly for sagittal and nonfrontal perspectives [35], which our per-placement results quantify directly. The framework also indicated that complex multiocular setups, like our multiocular example (DLT-MediaPipe), might not improve accuracy or occlusion robustness relative to modern monocular setups. While Neupane et al [21] report that multiocular systems can improve accuracy, Nogueira et al [50] also noted their sensitivity to occlusion. More recent multiocular HPE models that achieve a significantly increased spatial accuracy (MPJPE: 7.9 mm [58]) estimate the keypoints directly in 3D space rather than reconstructing 3D poses from 2D keypoints using, for example, DLT [50]. However, this often comes at the cost of real-time performance. Consequently, the added complexity and costs of multiocular setups may not justify modest gains in accuracy, for example, in telerehabilitation applications [21,50].

### Skeleton Morphing: Ensuring Validity in Interdataset Comparisons

A key framework component is the morphing model that is designed to align keypoint annotations across datasets and which we validate on the MediaPipe-to-Plug-in Gait mapping using controlled synthetic offsets. While its application enhances the validity of comparative analyses and benchmarking, due to dataset-dependent keypoint variations, prior studies did not apply this step. However, similar keypoint morphing methods have been developed, focusing primarily on training models across multiple datasets with different keypoint definitions [23,36,43]. Rapczyński et al [23] achieved a~37% improvement in MPJPE using linear methods to adjust for keypoint offsets and normalize scale across datasets before training. Although demonstrated to be effective, this linear approach cannot adequately capture nonlinear relationships between different keypoint annotation methods. However, Rapczyński et al [23] approach does not require a large dataset for the initial morphing model training, akin to that of Wandt et al [43], who used only one participant. We did not investigate how different training dataset sizes affect our morphing model’s performance, but a smaller subset may suffice, given the similarity of our model’s architecture to that of Wandt et al [43].

### Evaluation: Proposed Metrics and Thresholds in Clinical Settings

Beyond the MPJPE common in engineering benchmarks, clinical use demands domain-specific criteria across 3 dimensions: spatial accuracy, spatiotemporal consistency to capture movement dynamics, and latency for real-time feedback. We consider each in turn.

### Spatial Accuracy Requirements

As expected, AE (MPJAE) was less sensitive to global positional inaccuracies than MPJPE because joint angles depend on relative segment orientations rather than absolute keypoint positions.

However, while the approach used to calculate the joint angles as a relative difference from a participant-specific T-pose is simple and consistent, it may not replicate the physiotherapists’ by-eye judgment method. Thus, Mercadal-Baudart et al [59] proposed projecting the respective body segments onto a principal anatomical plane before calculating joint angles. This better simulates clinical assessments but only allows single-plane joint angle analysis.

To be able to place the observed MPJAE of 6 (SD 8) degrees into perspective, previous studies suggest that errors above 5 degrees might be misleading for clinical applications [19]. On the other hand, physiotherapists can only discern joint angles of >12 degrees in the visual assessment of single-plane movements [60]. The marker-based ground-truth is itself imperfect, with reported AEs from marker placement (<10 degrees) [61], soft-tissue artifacts (<3 degrees) [12], and joint-center estimation (<3 degrees) [62]. As HPE training data are often marker-derived, these errors may propagate into HPE estimates.

Finally, the framework’s spatial accuracy results were comparable with previous studies evaluating MediaPipe [59,63] and fall within the range recently reported for newer monocular estimators benchmarked against marker-based capture on clinical exercises, where per-joint position errors spanned roughly 72‐122 mm in the image plane and knee-flexion errors 9‐22 degrees [34]. This indicates that the limits of our framework are not specific to the exemplar model but characteristic of the current generation of markerless estimators.

### Spatiotemporal Consistency Requirements

Accurately estimating movement velocity and acceleration based solely on single-sensor position data is, in general, challenging. Inherent sensor noise and variability propagate and amplify during velocity and acceleration calculation, reducing the validity of such measurements [64]. Smoothing the position data beforehand may mitigate this, but its effectiveness depends on the chosen smoothing method and may hide relevant sensor noise or spatiotemporal inaccuracies. Thus, such metrics (like the MPJAVE) should be rather interpreted as a measure of spatiotemporal consistency or a measure of sensor jitter.

The sensitivity to sensor jitter of MPJAVE is evident in challenging scenarios, such as when occlusion is present, which leads to a higher frequency of insufficiently detected poses. The resulting noise from discontinuities (jumps) in the movement trajectories increases the overall MPJAVE slightly, underlining the necessity of smoothing velocity curves [64,65]. However, the observed MPJAVE of 4 (SD 8)°/s is acceptable, considering low velocities of reaching movements (75‐103°/s) [66] and during slow walking (85‐200°/s) [67].

The Pearson *r* supports this observation and shows a high spatiotemporal agreement of the movement trajectories between the gold-standard and HPE models. This agreement is consistent with previous studies [68].

### Latency Requirements for Real-Time Feedback

While for sports applications, motion capture systems typically sample between 50 and 250 Hz [69], slower rates (∼25‐40 Hz) can be permissible for motor rehabilitation applications due to slower gross movements [70,71]. In our experiment, MediaPipe (full model) maintained a stable rate of ∼40 Hz. However, the inference speed of HPE models depends significantly on the architecture of the model and the hardware used. Lighter models, which are at times faster, often compromise spatial accuracy [72].

### Proposed Benchmarking Thresholds

To provide a clinical assessment from these separate metrics, we propose an application-dependent interpretation of the metrics. An algorithm should satisfy spatial accuracy (MPJAE<5 degrees), followed by spatiotemporal accuracy (Pearson *r*>0.90). Finally, robustness is determined by the RBC effect size being lower than 0.3 across setup errors. This approach prevents a “good average score” from masking critical failures in specific clinical requirements and provides enough flexibility for different applications with different emphases.

### Framework Validity Across Rehabilitation Domains

Finally, the evaluation module assessed the framework’s validity across diverse movement tasks. Contrary to previous research emphasizing the necessity of training data including domain-specific movements [5,40], our observations suggest that, at least for this class of model, HPE trained on large and diverse general datasets can generalize well across diverse clinically relevant movement types, a finding that should be confirmed on additional models. While whole-body standing movements showed the highest spatial errors and sitting movements the lowest, the differences between movement types and monocular and multiocular setups remain negligible.

The joint-wise analysis exhibited decreased spatial accuracy of the left body side. As the simple movements were predominantly performed with the left body side, this naturally showed higher spatial errors than the static right side. Consequently, when assessing joint-wise spatial errors, the specific movement task and lateral asymmetries need to be taken into consideration. Additionally, distal joints were more vulnerable to spatial errors due to their increased flexibility and range of motion [73].

### Practical Considerations

Clinical deployment also requires attention to computational scalability. The evaluated model runs in real time on a standard workstation, and individual components can be swapped as HPE models evolve, so throughput depends on the chosen model rather than the framework itself. Patient-population validation and workflow integration are addressed under the Limitations section.

### Limitations

A few limitations of this study must be acknowledged. First, while our dataset covers a wide range of relevant movements for a relatively diverse population, it does not include pathological movements or anthropometric deviations. Although our results indicate that HPE models accurately estimate joint center locations across various movements, pathological movement patterns may affect accuracy, especially when mobility aids are used (eg, crutches, orthotics, and prosthetics) [74].

Second, this proof-of-concept applies the framework to a single exemplar model (MediaPipe). Because the framework is model-agnostic, benchmarking additional architectures, including those with different joint topologies, is a direct application of the pipeline rather than a change to it and is planned for future work. We note that the morphing architecture follows methods (Wandt et al [43], Rapczyński et al [23], and Sárándi et al [36]) already shown to generalize across datasets with differing skeleton formats.

Finally, regarding robustness, the current framework uses standard augmentations that simulate environmental noise. While sufficient for this proof-of-concept, they do not fully replicate complex semantic occlusions found in clinics. In addition, the framework’s modular design facilitates future integration of realistic 3D augmentations, such as inserting virtual objects (eg, wheelchairs) or persons (eg, therapists), to test scenario-specific distractions.

### Conclusions

This study establishes a technical foundation for the standardized evaluation of markerless tracking in clinical rehabilitation. By validating a modular pipeline, comprising augmentation, pose estimation, skeleton morphing, and evaluation modules, we have provided a transparent method for stakeholders in health care to assess the feasibility of real-time 3D HPE algorithms for their specific applications.

Applying this framework to a popular, accessible real-time algorithm indicated that the evaluated model was robust to environmental lighting variation and performed consistently across the tested movement set in healthy participants. However, the framework effectively identified critical operational limitations: specifically, occlusion, poor multiocular calibration, and side-facing camera placements were found to degrade tracking accuracy significantly. These findings highlight that while current algorithms are viable for general monitoring, strictly controlled setups are required to minimize line-of-sight obstructions.

Future research should extend this validation to pathological populations, integrate additional augmentations, and extend the set of postprocessing strategies and metrics (eg, Bland-Altman analysis) to further evaluate these systems. By providing a reproducible and domain-specific benchmarking approach, this framework facilitates effective integration of computer vision into motor rehabilitation workflows.

## Supplementary material

10.2196/91360 Multimedia Appendix 1 Methods and results, including the VizLab dataset description (marker set, red-green-blue camera and synchronization setup, experimental procedure, and data processing), metric equations, the skeleton morphing model architecture and training, the synthetic offset-recovery validation, and additional statistical tables (mean-per-joint position error, mean-per-joint angular velocity error, Pearson *r*, *P* values, and CIs).

## Abbreviations

AE: angular error
bl: back left
br: back right
DLT: direct linear transformation
fl: front left
fm: front middle
fr: front right
HPE: human pose estimation
MPJAE: mean-per-joint angular error
MPJAVE: mean-per-joint angular velocity error
MPJPE: mean-per-joint position error
PA-MPJPE: Procrustes aligned mean-per-joint position error
RBC: rank-biserial correlation
ReLU: rectified linear unit
RGB: red, green, blue-depth
RQ: research question
sl: side left
TTL: transistor-transistor logic
