# Supplementary material for: A Benchmarking Framework for Real-Time 3D Pose Estimation in Motor Rehabilitation: Development and Proof-of-Concept Study
Source: JMIR Rehabil Assist Technol. 2026 Nov 9;13:e91360. doi: 10.2196/91360 (PMC13747219; doi:10.2196/91360)
Supplement: Supplementary file 1 [file rehab-v13-e91360-s001.pdf]

A Benchmarking Framework for Real-Time 3D Pose Estimation in Motor Rehabilitation: Development and Proof-of-Concept Study

(REDACTED)

(REDACTED) Dataset

The capture space at the **(REDACTED)**, measured 5.74 x 5.4 meters, with a central movement area of 1.6 x 1.6 meters where the participant performed the movements (Figure 1). The laboratory had no windows, and its floor and walls were blue.

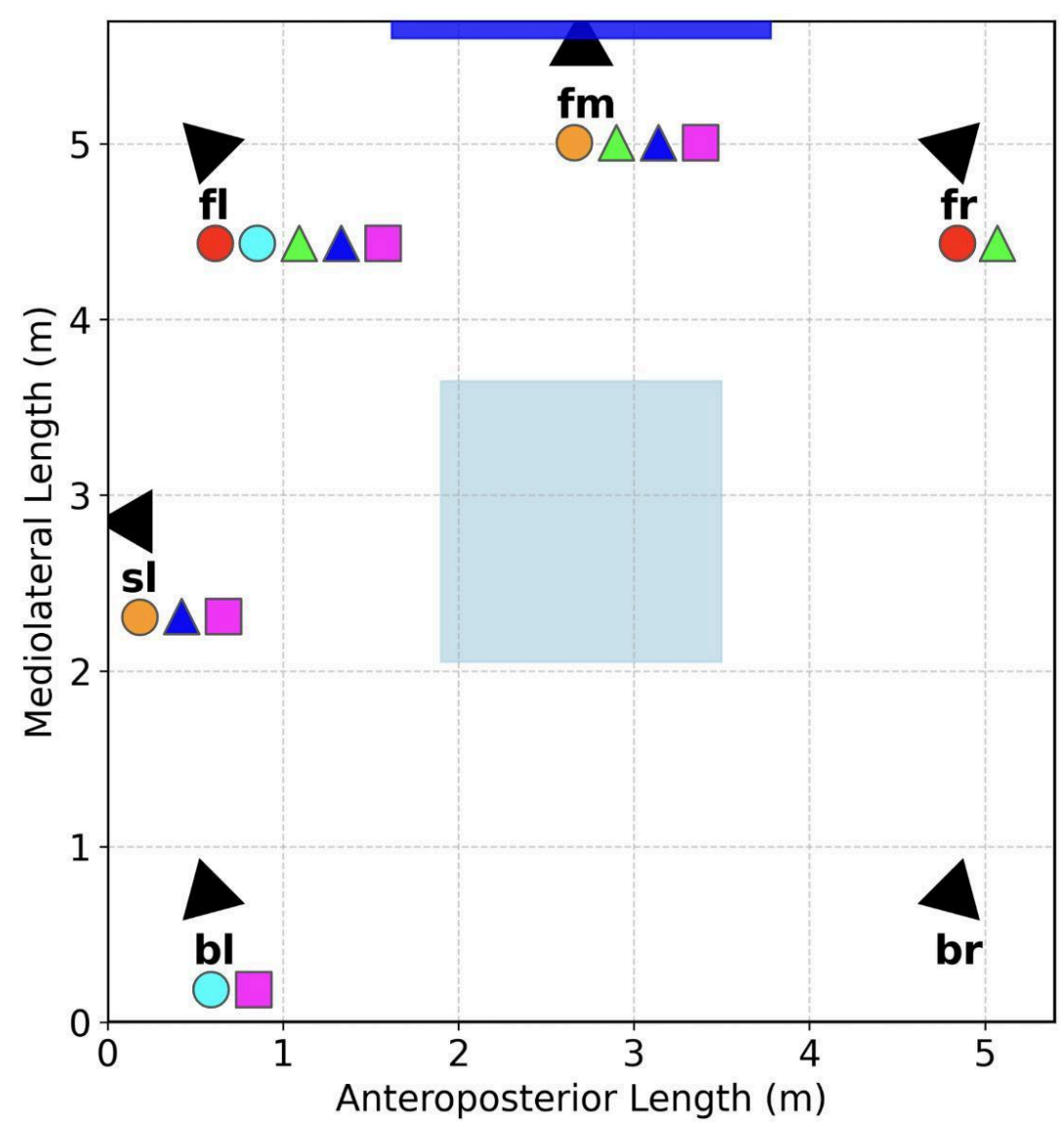

Figure 1. Top-down view of the laboratory setup, with the respective RGB video camera positions, instruction screen (blue), and movement area (light blue). The abbreviations indicate the camera positions as follows: Front-left (fl), front-middle (fm), front-right (fr), sl (side-left), back-left (bl), and back-right (br). Below each camera ID, colored dots indicate two-camera setups (fl-fr, fl-bl, fm-sl), triangles three-camera setups (fl-fm-fr, fl-fm-sl), and rectangles four-camera setups (fl-fm-bl-sl).

Movements

The movement sequences can be categorized as planar movements of left-sided body segments (upper/lower body) and whole-body movements while standing or sitting. Whole-body movements were expected to be more challenging for HPE models due to joint occlusion. Each movement was repeated ten times. A detailed list of the movements is displayed in Table 1.

Table 1. Categorization of performed movements.

Movement Type	Body Segment	Movement Sequence
**Planar Upper Body**	Shoulder	Flexion/Extension
		Abduction/Adduction
		Inward/Outward Rotation
	Elbow	Flexion/Extension
**Planar Lower Body**	Hip	Flexion/Extension
		Abduction/Adduction
		Inward/Outward Rotation
	Knee	Flexion/Extension
**Whole-Body Standing**	Whole Body	Forward Lunge
		Deep Squat
		Arm/Leg Crossover
		Trunk Rotation
		Step Sideways
**Whole-Body Sitting**	Whole Body	Sit-to-Stand/Stand-to-Sit
		Step Sideways
		Heel Raise
		Knee Extension

Marker-based Motion Capture

The baseline employed marker-based motion capture, which is considered the current gold-standard method. Eight ceiling-mounted cameras, consisting of 1x Oqus 300, 2x Oqus 310, and 5x Qualisys Opus 500+ (Qualisys AB, Stockholm, Sweden), were used. The data acquisition was facilitated by the Qualisys Track Manager (QTM) software at a sampling rate of 100 Hz. After the anthropometric measurements, an investigator with expertise in marker-based motion capture applied reflective markers with a diameter of 14 mm. The markers were placed according to Vicon's Plug-in Gait model. This marker set was chosen due to its widespread use in clinical practice and its modification to focus on the important segments/joints relevant for motor rehabilitation and alignment with current HPE models. A detailed description of modified marker placement is illustrated in Figure 2.

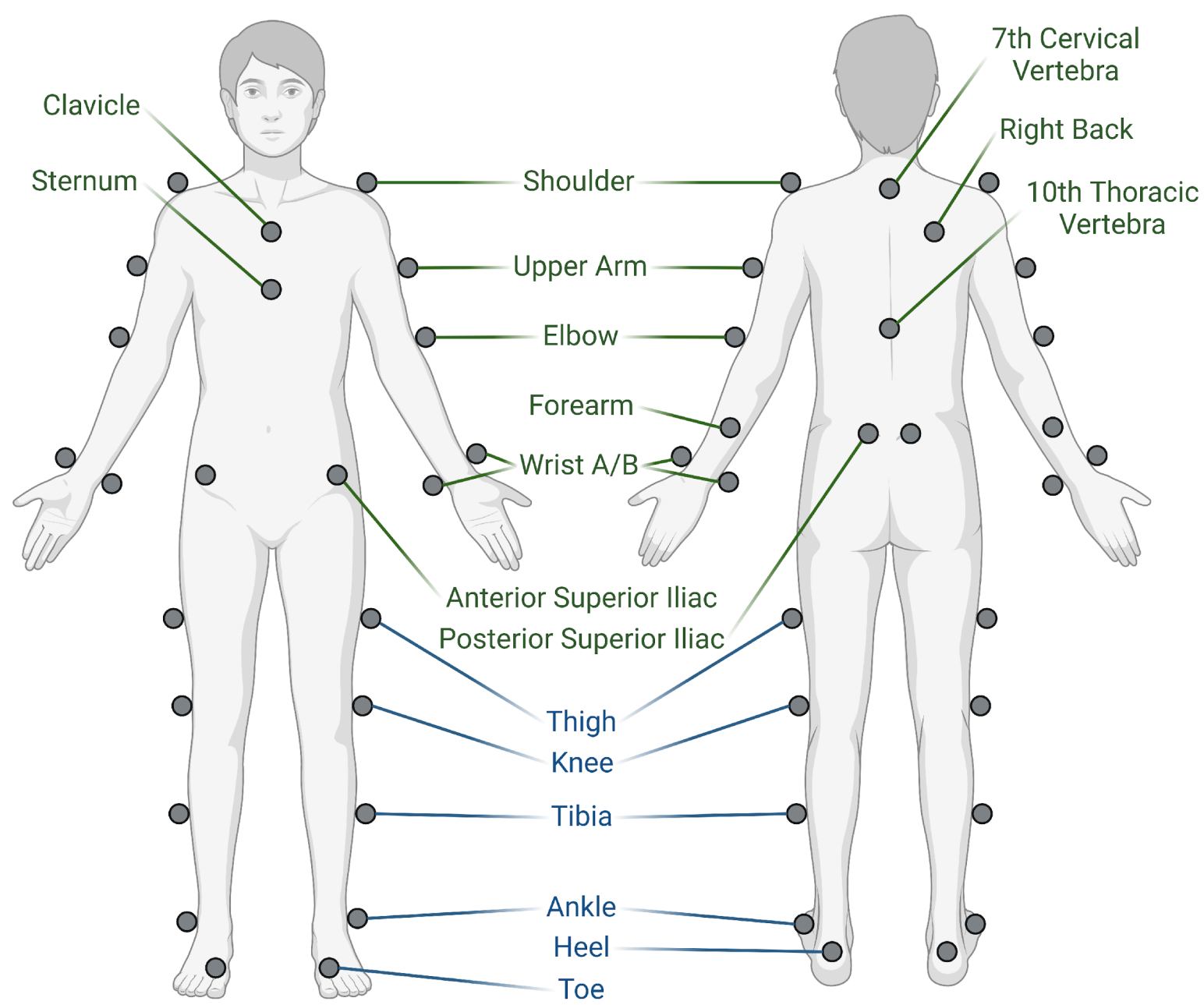

Figure 2. Modified marker placement based on Vicon's Plug-in Gait model [1]. Created in BioRender by Lorenz, E. (2025).

RGB Cameras

Video data was recorded using six FLIR Blackfly S (FLIR Systems Inc., OR, USA) at a sample rate of 25 Hz and a resolution of 1280 x 1024 px. The cameras were mounted on a stand at a 1-meter height (approx. desk height) and approximately 3 meters from the center of the room (Figure 1).

Movement Instruction Display

The movement instructions were displayed on a 75" LCD screen 2.22 meters from the center of the room in front of the participants. Unity (Unity Technologies, CA, USA) was used as presentation software. Upon every displayed movement iteration start and stop, an event trigger was sent via LabStreamingLayer (LSL) to facilitate movement segmentation during post-processing [2].

Synchronization

The temporal synchronization of both camera systems, namely the motion capture and RGB cameras, was achieved through a transistor-transistor-logic (TTL) signal. The Qualisys system operated as master, transmitting a TTL signal to the FLIR cameras upon shutter opening, triggering their shutter opening, and thus capturing a frame with a minimal temporal offset of approximately 14 microseconds. The LSL LabRecorder was employed to record the marker positions, camera frame number, and movement event markers [3]. This tool records and adjusts each data stream's timestamps to a common reference clock. A customized version of the Qualisys Lab Streaming Layer App client [4] captured the marker

positions. Concurrently, a custom Python script saved the corresponding video frames and transmitted the respective frame number via LSL upon receiving the video frame.

Experimental Procedure

Before the arrival of the participant, the marker-based system was calibrated. After briefing the participant on the experimental procedure and obtaining their consent, their anthropometric measurements were documented. Then, the markers were attached as mentioned, and the participant was positioned within the central movement area. A movement sequence was presented on the LCD screen three times, with the participants encouraged to replicate the movement to familiarize themself with the movements. After a short break, the movement sequence was displayed ten times, and the participant was instructed to perform it synchronously with the displayed movement.

Dataset Data Processing

The following chapter describes the post-processing of the various data streams recorded.

First, the motion capture data recorded with QTM was visually inspected in QTM. Missing or false marker labels were corrected, and an investigator with expertise in marker-based motion capture semi-automatically filled out gaps in the marker trajectories.

Vicon Nexus v. 2.15.0.145908h x64 (Vicon Motion Systems Ltd, UK) was used for further processing and modeling. First, the subject marker positions were scaled to a static skeleton using a T-Pose sequence. Then, the static PlugIn Gait model was used to calculate subject-specific offset based on the calibrated skeleton. Next, a second round of automatic and manual gap-filling was applied using kinematic and pattern-based algorithms. Then, the data was smoothed using a Butterworth low-pass filter (4th order, zero-lag, cut-off: 6 Hz), and the dynamic PlugIn Gait model was applied to calculate kinematic (and kinetic) outputs.

Using MATLAB v. R2023a (The MathWorks, Inc., Massachusetts, USA), customized scripts were developed to process the data further. A first script verified the resulting files from the previous processing steps. A second script then aligned the output of models with the temporally synchronized motion capture data recorded with the LSL LabRecorder based on the smallest Euclidean distance between both data streams. Thus, the processed motion capture stream is now temporally aligned with the RGB camera stream and movement event markers. The last script segmented the movement and video data according to the respective movement sequences.

Metrics

This section gives the formal definitions of the metrics summarized in the main text (Table 2). For each metric, we report its purpose, the domain it evaluates (spatial accuracy, spatiotemporal accuracy, or real-time capability), and its equation. Notation is consistent throughout: p and $\widehat{p}$ denote ground-truth and predicted keypoints, R and $\widehat{R}$ the

corresponding joint angles, N_J the number of joints, T the number of frames in the time window, and N_F the total number of frames.

Table 2. Equation of used metrics.

Metric	Description	Equation
**Spatial accuracy**		
**MPJPE**	The mean-per-joint position error (MPJPE) calculates the Euclidean distance between the predicted keypoints $\hat{p}$ and their respective ground truth p over all frames F and joints J to evaluate the spatial accuracy of the model in millimeters. Lower is better.	$\mathrm{MPJPE} = \frac{1}{N_J} \sum_{j=1}^{N_J} \lVert p_j - \hat{p}_j \rVert_2$
**MPJAE**	The mean-per-joint angular error (MPJAE) calculates the average deviation between predicted $\hat{R}$ and the ground truth R joint angles across all axes a and joints J. The respective angles are calculated as the deviation from the T-pose [4]. Lower is better.	$\mathrm{MPJAE} = \frac{1}{3 \cdot N_J} \sum_{j=1}^{N_J} \sum_{a \in \{x,y,z\}} \left\lvert R_{j,a} - \hat{R}_{j,a} \right\rvert$
**Spatiotemporal accuracy**		
**MPJAVE**	The mean-per-joint angular velocity error (MPJAVE) calculates the average deviation between the smoothed (Savitzky-Golay, $window\ size = 2\,\frac{1}{T} + 1$, $order = 3$) predicted joint angle $\hat{R}$ and ground truth R over a time window T, based on the temporal derivative of the joint angle. Lower is better.	$\mathrm{MPJAVE} = \frac{1}{N_J} \sum_{j=1}^{N_J} \frac{1}{T-2} \sum_{t=2}^{T-1} \left\lvert \frac{\Delta R_{j,t} - \Delta \hat{R}_{j,t}}{2\Delta t} \right\rvert$
**Pearson *r***	The mean-per-joint Pearson correlation coefficient (*r*) measures spatiotemporal similarity ρ between the predicted joint trajectories $\hat{p}$ and ground truth p. Closer to 1 is better.	$\mathrm{PCC} = \frac{1}{N_J} \sum_{j=1}^{N_J} \rho(p_j, \hat{p}_j)$
**Real-time capability**		
**Inference time**	Inference time reflects the model's real-time applicability averaged over all frames F. Lower is better.	$\text{Inference Time} = \frac{1}{N_F} \sum_{f=1}^{N_f} t_f$

Skeleton Morphing Module

Architecture of Morphing Model

The neural network first upscaled the 48-dimensional input - representing 16 keypoints in 3D space - into a higher-dimensional latent space with 2048 dimensions via a linear layer. The upscaled representation is then processed through two residual blocks, maintaining the data's original structure while learning meaningful features. Subsequently, a second linear layer transformed the data back to the original 48-dimensional space. Lastly, the learned inter-dataset keypoint relationships were added to the original input pose via a residual connection, morphing the HPE-keypoint to the Qualisys-based keypoints. The model applied a dropout to prevent overfitting and utilized Leaky ReLU activation to introduce non-linearity in the transformations and mitigate neuron inactivity.

Dataset for Testing and Training

The morphing model was trained on a sample of 15 participants (six females), with a mean age of 32 years (Standard Deviation (SD) 11), a mean height of 168 cm (SD 10), and a mean weight of 75 kg (SD 13). The data was split using a custom cross-validation strategy (similar to leave-one-out cross-validation). Each fold was validated on data from two unseen participants, preventing the model from learning participant-specific features. To reduce redundancy in keypoint positions, only every 25th sample (1 sec steps) was used, and the order of the data was randomized for each fold. The trained morphing model was finally tested on two unseen participants. The participant data used for training the morphing model was excluded from the subsequent HPE model evaluation pipeline to prevent data leakage.

Training and Hyperparameter Optimization

The morphing model was trained using the AdamW optimizer, combined with a learning rate scheduler that reduced the learning rate by a factor of 0.1 whenever performance stagnated for ten epochs, as determined by a dynamic threshold. Two rounds of hyperparameter optimization were done using `wandb` platform [5]. The first round provided a rough overview of the relevant hyperparameter ranges, while the second round focused on fine-tuning key hyperparameters using Bayesian hyperparameter optimization, minimizing the Procrustes Aligned Mean Squared Error (Procrustes-MSE) of the morphed HPE-model keypoints towards the Qualisys-based keypoints. Table 3 summarizes the optimized parameters and their optimal values after 23 sweeps, at which their loss converged and computational time limits were met (Figure 3).

Table 3. Optimized hyperparameters, their optimization range, and their final optimum.

**Hyperparameter**	**Range**	**Optimum**
**Batch Size**	32, 64, 128	64
**Epochs**	60, 80	80
**Learning Rate**	0.00005, 0.0001, 0.001	0.00005
**Weight Decay**	0.0001, 0.001, 0.1	0.001
**Layer size**	1024, 2048	2048
**Dropout Rate**	0, 0.1, 0.2	0.2

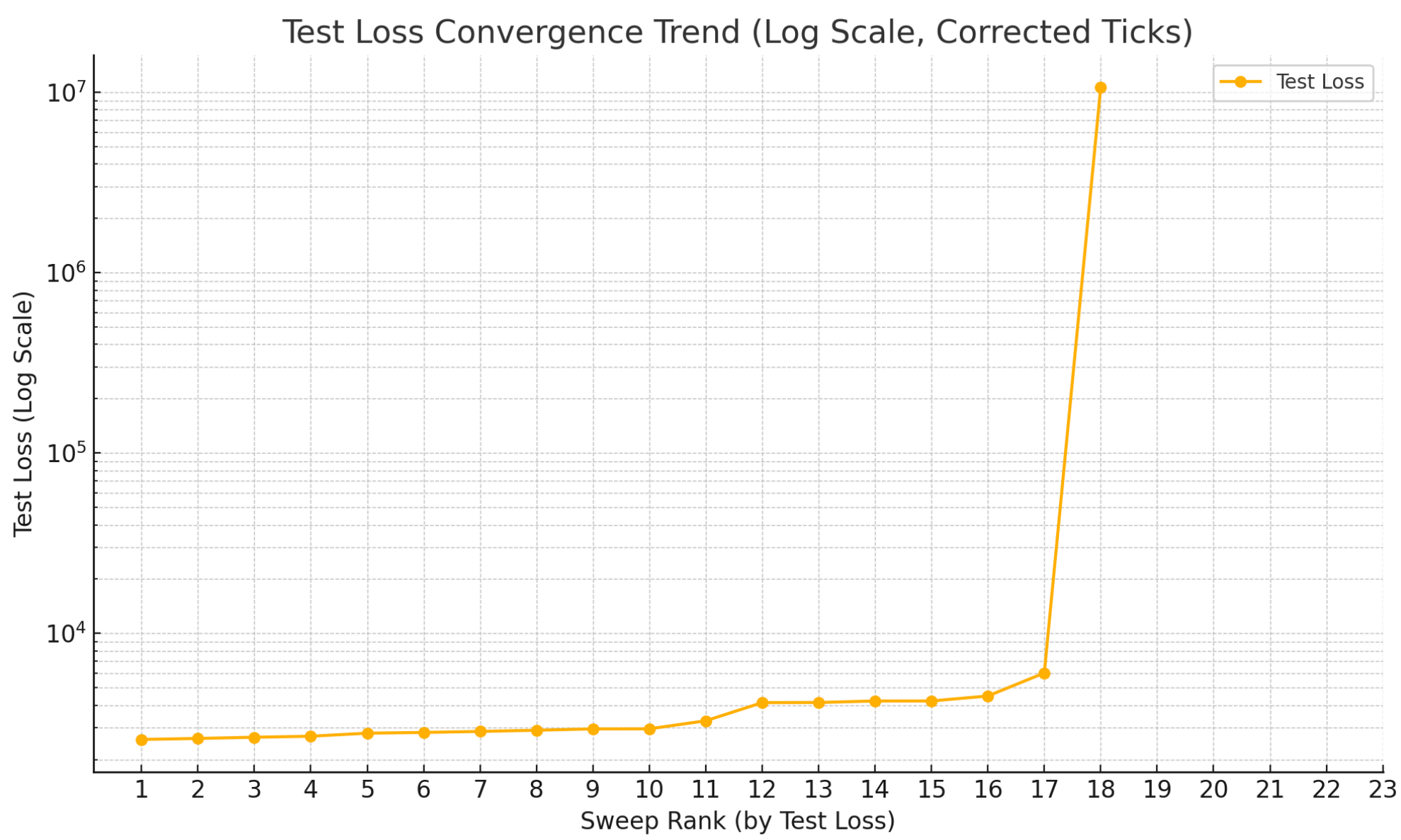

Figure 3. Test loss convergence trends for different hyperparameter optimization sweeps, represented by the Procrustes-aligned Mean Per Joint Position Error (P-MPJPE) on a logarithmic scale.

Validation Method of Morphing Model

Since this offset is unknown for real data, it cannot be separated from actual tracking errors by direct measurement. We therefore tested the model on synthetic data with a known offset and known noise, so that both could be quantified separately.

We generated synthetic poses by adding a known systematic offset, which the morphing model should remove, and independent random noise, which it should preserve, to the ground-truth (marker-based) poses. The offset consisted of a constant (linear) and a movement-dependent (nonlinear) component, motivated by the two ways a skin-surface-based HPE keypoint is expected to diverge from a body-internal, marker-based joint center [6]. The linear component was a fixed per-joint translation along the outward

radial direction $\hat{r}_j$ (perpendicular to the joint's segment, pointing from the internal joint center toward the skin surface), scaled by a = 40 mm and a distality weight w_j, assigning larger offsets to distal joints, consistent with the discrepancy reported for HPE models [7]. The nonlinear component applied the same direction scaled by the joint's flexion $\tilde{\varphi}$ (standardized per joint), with b = 30 mm. This component approximates that soft-tissue movement around a joint, and therefore the surface-to-center offset, increases with joint flexion [6]. The noise was drawn independently per joint, axis, and frame, and therefore cannot be removed by any pose-dependent mapping. The offset was added in the ground-truth frame so that the subsequent Procrustes alignment, which removes differences in rotation, translation, and scale, would not remove it.

We evaluated two variants. In the controlled variant, the offset was added to the ground truth ($S = G + \Delta + \varepsilon$), and in the realistic variant, to the Procrustes-aligned HPE (Mediapipe) output ($S = \tilde{H} + \Delta + \varepsilon$). For each variant, we trained a separate morphing model, identical to the one described above, on 13 participants and tested it on 2 held-out participants, under three regimes: linear only, nonlinear only, and combined.

We report the following measures, per joint and averaged over the held-out participants. Offset recovery, computed on the noise-free stream, $S_{clean} = reference + \Delta$, is the fraction of the known offset removed:

$$recovery_j = 1 - \frac{mean_f \lVert M(S_{clean}) - G \rVert_j}{mean_f \lVert S_{clean} - G \rVert_j}$$

Noise preservation is the ratio of noise spread after to before morphing, $\eta_j = std(M(S_{noisy}) - M(S_{clean}))_j \,/\, std(\varepsilon)_j$, where $\eta \approx 1$ indicates the noise passed through unchanged and $\eta < 1$ indicates that it was suppressed. We additionally compared the model against a per-joint linear baseline method (a least-squares affine map fitted on the training participants) [7] and report PA-MPJPE before and after morphing. As in the main analysis, the large frame count makes significance testing uninformative, we interpret effect sizes and absolute differences rather than P values. Furthermore, as consecutive frames are correlated in time, the effective sample is smaller than the frame count, and the confidence intervals should be read as optimistic.

Validation Results of the Skeleton Morphing Model

This section reports the synthetic-offset validation described above, quantifying how much of a known joint-definition offset the morphing model removes and how much of the injected noise it preserves. Results are given per offset regime (linear, nonlinear, combined) for both the controlled and realistic variants, on the two held-out participants, and are compared against a per-joint linear baseline (Figure 4).

In the controlled variant, the morphing model recovered the constant (linear) offset well (93.3%), similar to the linear baseline method (98.7%). For the nonlinear offset, which the linear baseline method cannot fully address, the morphing model recovered 65.0% against

21.0% for the baseline method, and for the combined offset, 81.9% against 65.6% (Figure 4). In absolute terms, PA-MPJPE fell from 33.33 (SD 17.25) mm to 10.65 (SD 5.54) mm for the combined offset and from 16.56 (SD 11.38) to 9.51 (SD 5.09) mm for the nonlinear offset, with near-ceiling effect sizes (RBC = 1.00 and 0.99). The largest per-joint effects occurred at the distal joints that flexed most during the mainly left-sided movements, for example, the left shoulder (RBC = 0.85), elbow (0.63), and wrist (0.61). The noise-preservation ratio stayed between 0.97 and 1.00 across all conditions, indicating that the model removed the systematic offset without suppressing tracking noise. In the realistic variant, PA-MPJPE fell from about 104 to 35 mm across all regimes (noise preservation 0.97–0.98). Because real tracking error is also present and unknown here, we report these as a robustness check rather than exact recovery.

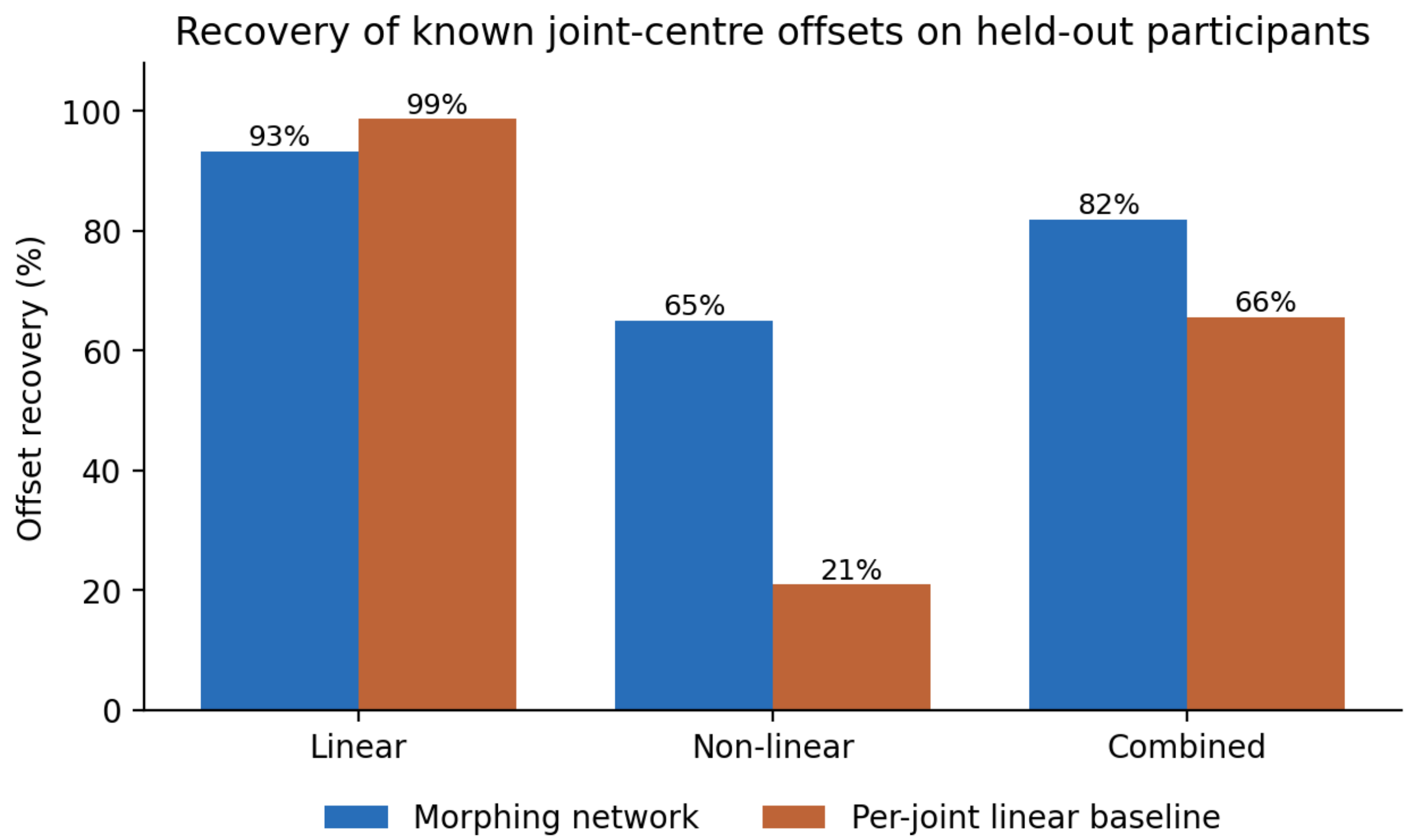

Figure 4. Recovery of known joint-center offsets by the morphing model and a per-joint linear baseline on held-out participants, for three offset regimes. Recovery is the fraction of the injected offset removed (100% = fully removed). The baseline matches the morphing model on the constant (linear) offset but recovers little of the pose-dependent (nonlinear) offset; the morphing model recovers most of it. The noise-preservation ratio was 0.97–1.00 in all regimes (not shown), indicating tracking noise was retained rather than suppressed.

Additional Results of Proof-of-Concept Evaluation

Benchmarking Robustness to Simulated Clinical Setup Errors

This section supplements the main-text setup-error analysis with the secondary metrics. Table 4 reports MPJPE, MPJAVE, and Pearson r for each augmentation relative to baseline, and Table 5 gives the corresponding P values and confidence intervals for monocular and multiocular setups on the default camera positions. As in the main analysis, the large frame count (N=127,828) makes significance testing highly sensitive, so P values are reported for completeness, and the effect sizes in the main text carry the interpretation.

Table 4. Mean-per-joint Position Error (MPJPE), Mean-per-joint Angular Velocity Error (MPJAVE), and Pearson r across simulated setup errors for monocular and multiocular Human Pose Estimation setups. Results are averaged over all joints and movements using default camera positions (mono: front-middle; multi: front-left/front-right) on 4 healthy participants (N=127,828 total frames).

**Setup error**	**MPJPE[a] [mm]**		**MPJAVE[b] [°/s]**		**Pearson *r***	
	**Mono**	**Multi**	**Mono**	**Multi**	**Mono**	**Multi**
**Background**	33 (27)	45 (56)	4 (8)	5 (11)	0.95	0.90
**Defocus**	30 (23)	35 (24)	4 (8)	4 (10)	0.96	0.94
**Occlusion**	**35 (36)**	45 (53)	**4 (8)**	5 (9)	**0.93**	0.89
**Underexposure**	30 (22)	34 (23)	4 (8)	4 (10)	0.96	0.94
**Decalibrated**	-	**137 (92)**	-	**8 (16)**	-	**0.65**
**Desynchronized**	-	34 (24)	-	4 (10)	-	0.94

[a]*Mean-per-joint position error*

[b]*Mean-per-joint angular velocity error*

Table 5. P value and Confidence Interval (CI) of Mean-per-joint Angular Error (MPJAE) for setup errors in monoocular and multiocular Human Pose Estimation setups. The analysis compares augmented conditions to the baseline (no augmentation) for all movements in 4 healthy participants (N=127,828).

**Setup Error**	***P* value (CI[a])**	
	**Mono**	**Multi**
Background	< .001<br>(0.27, 0.32)	< .001<br>(0.93, 1.00)
Defocus	< .001<br>(0.05, 0.10)	< .001<br>(0.05, 0.10)
Occlusion	< .001<br>(0.71, 0.77)	< .001<br>(1.50, 1.57)
Underexposure	< .001<br>(−0.09, −0.04)	0.511<br>(−0.04, 0.01)
Decalibrated	-	< .001<br>(7.77, 7.84)
Desynchronized	-	0.973<br>(−0.02, 0.02)

[a]Confidence interval

Impact of Spatial Configuration Across Camera Placements

This section provides the statistical support for the camera-placement findings in the main text. Table 6 reports MPJPE, MPJAVE, and Pearson r for every monocular and multiocular placement on non-augmented inputs, and Table 7 gives the P values and bootstrap confidence intervals for each placement relative to its default (mono: front-middle; multi: front-left/front-right). Bias is quantified as the bootstrap mean difference with its 95% CI.

Table 6. Mean (standard deviation) of Mean-per-joint Position Error (MPJPE), Mean-per-joint Angular Velocity Error (MPJAVE), and Pearson r for all camera placements across all movements. Results are shown for monocular and multiocular setups on non-augmented inputs from 4 healthy participants (N=127,828 total frames).

**Placement**	**MPJPE[a] [mm]**	**MPJAVE[b] [°/s]**	**Pearson *r***
**fl**	32 (22)	4 (8)	0.96
**fm**	**30 (22)**	**4 (8)**	**0.96**
**fr**	31 (23)	4 (8)	0.95
**br**	40 (30)	5 (11)	0.94
**bl**	40 (27)	5 (10)	0.93
**sl**	47 (46)	6 (12)	0.92
**fl, fr**	34 (24)	4 (10)	0.94
**fl, bl**	36 (24)	4 (9)	0.94
**fm, sl**	45 (39)	5 (11)	0.93
**fl, fm, fr**	**33 (22)**	**4 (9)**	**0.95**
**fl, fm, sl**	40 (30)	4 (10)	0.94
**fl, fm, bl, sl**	39 (28)	4 (9)	0.94

[a]*Mean-per-joint position error*

[b]*Mean-per-joint angular velocity error*

Table 7. P value and Confidence Interval (CI) of Mean-per-joint Angular Error (MPJAE) for all camera placements relative to default positions. The analysis compares different monoocular and multiocular placements to their respective default setup (mono: front-middle; multi: front-left/front-right) on non-augmented inputs from 4 healthy participants (N=127,828 total frames).

**Cameras**	***P* value (CI[a])**
fl	0.013<br>(−0.05, −0.01)
fr	< .001<br>(0.07, 0.12)
br	< .001<br>(1.74, 1.80)
bl	< .001<br>(1.62, 1.67)
sl	< .001<br>(2.14, 2.20)
fl, bl	< .001<br>(0.35, 0.40)
fm, sl	< .001<br>(1.03, 1.09)
fl, fm, fr	< .001<br>(−0.22, −0.17)
fl, fm, sl	< .001<br>(0.29, 0.34)
fl, fm, bl, sl	< .001<br>(0.12, 0.17)

[a]Confidence interval

Influence of Movement Type on Accuracy

This section reports how the movement category affected accuracy. Table 8 gives MPJPE, MPJAVE, and Pearson r for upper-body, lower-body, and whole-body standing and sitting movements, and Table 9 reports the pairwise post-hoc comparisons (mean difference, t, and Hedges g) between categories, for monocular and multiocular setups on non-augmented inputs from the default camera positions.

Table 8. Mean (standard deviation) of Mean-per-joint Position Error (MPJPE), Mean-per-joint Angular Velocity Error (MPJAVE), and Pearson r across different movement categories. Results are based on non-augmented inputs from the default camera positions in 4 healthy participants, demonstrating the influence of movement type on pose estimation accuracy.

**Movement Type**	**MPJPE[a] [mm]**		**MPJAVE[b] [°/s]**		**Pearson *r***		**n**
	**Mono**	**Multi**	**Mono**	**Multi**	**Mono**	**Multi**	
**Upper**	30 (24)	34 (25)	4 (10)	4 (9)	0.92	0.90	30,521
**Lower**	28 (19)	31 (22)	4 (8)	4 (9)	0.91	0.89	29,818
**Standing**	31 (24)	36 (23)	4 (8)	4 (8)	0.94	0.91	37,967
**Sitting**	**25 (17)**	**27 (18)**	**3 (9)**	**3 (8)**	**0.95**	**0.91**	29,522

[a]*Mean-per-joint position error*

[b]*Mean-per-joint angular velocity error*

Table 9. Posthoc comparisons of Mean-per-joint Angular Error (MPJAE) across movement categories in monocular and multiocular Human Pose Estimation setups: Mean difference (standard error), t-statistic, and Hedges g effect size. Differences between movement types were assessed using the Games-Howell test with Benjamini–Hochberg correction on nonaugmented inputs from 4 participants. All comparisons were significant (p<.05), except those marked [b], with the largest effects in bold.

**Comparison**	**Mean difference (SE[a]) [°]**	***t (df)***	**Effect size (Hedges *g*)**
**Monocular**			
**Standing/Lower**	0.20 (0.02)	8.58 (67,324)	0.07
**Standing/Sitting**	−0.53 (0.03)	−20.94 (63,118)	−0.16
**Standing/Upper**	0.08 (0.02)	3.62 (67,981)	0.03
**Lower/Sitting**	**−0.73 (0.02)**	**−29.24 (57,510)**	**−0.24**
**Lower/Upper**	−0.11 (0.02)	−4.99 (60,336)	−0.04
**Sitting/Upper**	0.62 (0.02)	24.50 (58,254)	0.20
**Multiocular**			
**Standing/Lower**	0.15 (0.03)	5.99 (63,823)	0.05
**Standing/Sitting**	0.49 (0.02)	19.78 (64,164)	0.15
**Standing/Upper**	**0.49 (0.02)**	**21.78 (68,451)**	**0.16**
**Lower/Sitting**	0.34 (0.03)	12.88 (59,310)	0.11
**Lower/Upper**	0.34 (0.02)	14.03 (57,488)	0.11
**Sitting/Upper[b]**	0.00 (0.02)	0.04 (57,689)	0.00

[a]*Standard error*

[b]*Non-signficant*

References

1. Vicon. Plug-in Gait Reference Guide. Vicon Motion Systems Limited; 2021. Available from: https://help.vicon.com/download/attachments/11250652/Plug-in%20Gait%20Reference%20Guide.pdf [accessed Jan 27, 2025]

2. Stenner T, Boulay C, Grivich M, Medine D, Kothe C, Herzke T, Grimm G, Biancarelli A, Mansencal B, Frey J, Powell S, Clisson P. sccn/liblsl: v1.16.0. Zenodo; 2022. Available from: https://zenodo.org/records/6387090 [accessed Nov 25, 2024]

3. Swartz Center for Computational Neuroscience. LabStreamingLayer. Swartz Center for Computational Neuroscience; 2023. Available from: https://github.com/sccn/labstreaminglayer [accessed Apr 25, 2023]

4. Batpurev T. Joint Angles Calculate. 2021. Available from: https://github.com/TemugeB/joint_angles_calculate

5. Biewald L. Experiment Tracking with Weights and Biases. CA, USA: Weights & Biases; 2020.

6. Benoit DL, Damsgaard M, Andersen MS. Surface marker cluster translation, rotation, scaling and deformation: their contribution to soft tissue artefact and impact on knee joint kinematics. J Biomech 2015;48(10):2124–2129.

7. Rapczyński M, Werner P, Handrich S, Al-Hamadi A. A baseline for cross-database 3D human pose estimation. Sensors 2021 May 28;21(11):3769.
